# Unification Theory of Optimal Life Histories and Linear Demographic Models in Internal Stochasticity

**DOI:** 10.1371/journal.pone.0098746

**Published:** 2014-06-19

**Authors:** Ryo Oizumi

**Affiliations:** Graduate School of Mathematical Science, University of Tokyo, Tokyo, Japan; University of South Australia, Australia

## Abstract

Life history of organisms is exposed to uncertainty generated by internal and external stochasticities. Internal stochasticity is generated by the randomness in each individual life history, such as randomness in food intake, genetic character and size growth rate, whereas external stochasticity is due to the environment. For instance, it is known that the external stochasticity tends to affect population growth rate negatively. It has been shown in a recent theoretical study using path-integral formulation in structured linear demographic models that internal stochasticity can affect population growth rate positively or negatively. However, internal stochasticity has not been the main subject of researches. Taking account of effect of internal stochasticity on the population growth rate, the fittest organism has the optimal control of life history affected by the stochasticity in the habitat. The study of this control is known as the optimal life schedule problems. In order to analyze the optimal control under internal stochasticity, we need to make use of “Stochastic Control Theory” in the optimal life schedule problem. There is, however, no such kind of theory unifying optimal life history and internal stochasticity. This study focuses on an extension of optimal life schedule problems to unify control theory of internal stochasticity into linear demographic models. First, we show the relationship between the general age-states linear demographic models and the stochastic control theory via several mathematical formulations, such as path–integral, integral equation, and transition matrix. Secondly, we apply our theory to a two-resource utilization model for two different breeding systems: semelparity and iteroparity. Finally, we show that the diversity of resources is important for species in a case. Our study shows that this unification theory can address risk hedges of life history in general age-states linear demographic models.

## Introduction

Environmental stochasticity is one of the problems which organisms face in their life schedule because it brings uncertainty to their maturity and reproduction timing. Demographers examine the effect of stochasticity on population dynamics by using linear demographic models (LDM), such as transition matrix models (TMM), integral projection models (IPM), and partial differential equations (PDE). As a consequence, they showed that stochasticity affects population growth rate negatively [1]–[6]. Empirical researchers are devoted to estimating the effect of external stochasticity on the life history of organisms [7]–[9]. However, there actually exists a twofold stochasticity in their models, that is, internal and environmental stochasticity (i.e., external stochasticities). For example, in TMM used by ecologists, the internal stochasticity yields a set of transition probabilities to other states and is generated by the dispersion of each individual life history, such as the dispersion of feed intake, genetic character, and size growth rate; whereas external stochasticity annually modifies the value of these transition probabilities. It is difficult to distinguish one stochastic effect from the other. Research on internal stochasticity is limited because the effect is different among formalizations of LDMs and we have not had the proper methods to analyze the effect of internal stochasticity on the dominant eiganvalue.

In a recent study, Oizumi and Takada (2013) obtained the methodology to show that internal stochasticity could increase and decrease population growth rates depending on the breeding system [10]. Additionally, the study provided a way to analyze the effect of the internal stochasticity on the population growth rate by using formulae of stochastic differential equations. Life history with internal stochasticity generates a population density function (population vector) that can be expressed by an age-size structured LDM. Then, the effect of internal stochasticity appears as a diffusion term in the LDM. The dominant eigenvalue of LDM is called intrinsic rate of natural increase, or fitness (which this paper uses) in theoretical demography, and is derived from the characteristic equation of the LDM, so called Euler–Lotka equation. The equation is composed of the breeding system, mortality, and the physiological growth process affected by internal stochasticity. In nature, it is considered that species control those three elements to become optimal in the habitat. Finding the optimal control is called “optimal life schedule problem” (OLSP) in theoretical biology. The analysis of LDM and OLSP was unified by Taylor 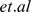 (1974) and Leon (1976) for deterministic life history via methods of dynamical programming [11], [12]. Oizumi and Takada (2013) extended the proof to a life history with stochastic growth via a novel method that is called path-integral formulation; besides, they showed by their method that internal stochasticity serves as a significant factor involved in persistence of species as well as external stochasticity [10]. Considering control of the stochasticity is hence concerned with not only various issues on conservation ecology, but evolution of life history. Internal stochasticity has a lot of ground to cover as mentioned above, even though their theory deal with optimal breeding timing in life history affected by an internal stochasticity only. To take account of multifaceted parts in life history, their theory should extend to multi–stage LDMs affected by several numbers of internal stochasticities and be able to analyze not only optimal control of reproductive timing but also of each transition rate. In this paper, we focus on unifying “Stochastic Control Theory” into multi–stage LDMs for that reason. This theory is extension of dynamical programming to control of stochastic process, i.e. we examine the theorem of Taylor 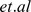 (1974), Leon (1976), Oizumi and Takada (2013) to adapt not only optimal reproductive timing but also the whole control of life history with internal stochasticity.

Stochastic control theory is normally used in control engineering and mathematical finance. In mathematical finance, it is applied to the analysis of optimal risk management in personal assets, where the optimal control determines utilization of investment between investment trust and loss insurance for the personal utility maximization [13], [14]. Mathematical economists define a function, called utility function, and search for the optimal control maximizing it. The function normally belongs to a class of concave functions and is assumed by it. Their convexity is a key point finding the optimal strategy because this property provides some extreme values related to the strategy. The idea of economists resembles that of an organism utilizing resources for its own maintenance and reproduction. In fact, mathematical biologists studying OLSP use functions, called reproductive value, similar to utility functions used by economists [15], [16]. Those functions are generally called objective functions in control theory. To unify OLSP with internal stochasticity into LDMs, an optimized objective function in biology must yield the maximum fitness. However, reproductive values which appear in their analysis are not always clear relation with the fitness. Studies of Taylor 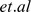 (1974) and Leon (1976) postulated only age–structured LDM and is obscure in LDMs incident to their objective function, respectively. Under internal stochasticity, Oizumi and Takada (2013) asserted that the objective function should be the Laplace transform of the expectation of the reproductive success (ERS), because it is generated by the Euler–Lotka equation in the age-size structured model. The function has the same meaning as the one used in Taylor 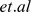 and Leon. Although Oizumi and Takada mentioned the relationship between path-integral formulation and other LDMs, they were not enough to elucidate the correspondence to all formulation of LDMs.

In this paper, we address control of a life history having 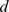-states affected by 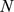 internal stochasticities as extension of the previous work [10]: besides, we show the relationship between path–integral formulation and other LDMs in the life history. We unify OLSP and LDMs at the life history via an optimized objective function derived from an important equation from control theory (Hamilton–Jacobi–Bellman equation). Applying our theory to analysis of a simple risk control problem (two-resource utilization model) in two distinct breeding systems, we discuss the meaning of the maximized objective function and the relationship between the optimal strategy and the structure of population.

## Analysis

### Configuration of individual life history

Several stochasticities in life history have been assumed by diffusion process such as migration of population [17], [18], size growth [10], [19], personal assets in human society as well [13], [20]. We assume to categorize them with effect of internal stochasticity and construct the configuration of two types of breeding systems (semelparity and iteroparity) as general stochastic control process in this section.

#### States transition processes

We consider individuals having 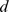-state 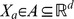 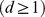 at age 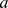, 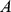 being the domain of each state. They are assumed to be fluctuated by the 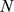 internal stochastisities. Then, the growth rate of each state is provided by the following Ito's SDE:
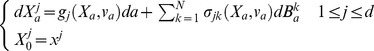
(1)where 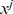 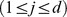 represents an initial state of 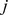-th state, 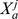. In this study, we assume all individuals have the same inital state 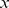. On the right-hand side, the first drift term represents deterministic rule of growth process of the element, whereas the second term represents fluctuation at 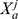, and 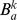 denotes an element of 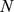-dimensional Brownian motion. We set
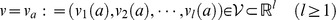
which represents a control vector and 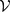 is a compact convex set of 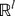.

#### Fertility function and breeding systems

We introduce a general fertility which 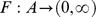 has integrability with respect to a probability measure 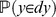 as follows:
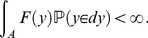
(2)Semelparous and iteroparous breeding systems are defined as types of fertility function.

In semelparous species, denoting mature state 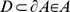 where 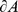 is boundary of 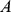 and the mature age 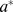 such that
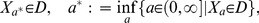
(3)the fertility, 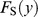, is defined by
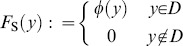
(4)where the fertility rate function satisfies 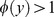 at a mature state 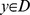. From biological point of view, 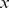 should be 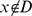. While, we define an iteroparous fertility function 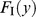 as continuous, and measurable function given by Eq.(2). Then, the species can reproduce at any time during their growth state.

We use fertility, Eq.(2), as a general breeding system if the analysis is common to semelparous and iteroparous breeding systems.

#### Mortality and survivorship

Semelparous and iteroparous species have different survivorship. We set a common mortality function, 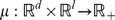, depending on 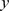 as follows:
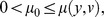
in both types of species. A general survivorship 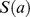 until age 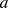 is written as
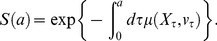
(5)


Since semelparous species die upon reproduction, we can write the survivorship as
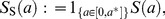
(6)by using an indicator function and the general survivorship, Eq.(5). While, we assume that iteroparous species have a limit of lifespan. Letting 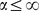 be the limit of lifespan, the iteroparous survivorship is written as
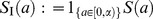
(7)If the maximum lifespan follows 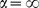, the species only die by accidental death and Eq.(7) corresponds to Eq.(5).

#### Difference of life history between semelparity and iteroparity

We here call Eqs.(1), (2), and (5) as life history. Then, we define a semelparous and an iteroparous life history following Eqs.(4) and (6), and 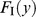 and Eq.(7) under the growth rates Eq.(1), respectively. In other words, we identify each life history with their difference in fertility and survivorship.

### LDM and objective function

In order to analyze that two life histories influence each population dynamics, we unify the individual life history into its population density function formalized by path-integral: after that, we demonstrate similarities among LDMs via the path-integral formulation in this section. In this study, we refer to this sort of population density function parametarized by time, age, and states as “population vector”.

#### Age-state structured model with internal stochasticity

Setting 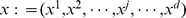 as an initial state vector of Eq.(1), we consider the following quantity:
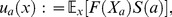
(8)where this function represents the expectation of the product of fertility and mortality at age 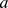, and we refer to it as an expectation of reproductive success (ERS) wich is identical to net reproduction function in demography [21]. The expectation in Eq.(8) is given by the probability measure which Eq.(1) generates. Oizumi and Takada showed, in 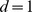 and 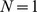, that a characteristic equation,
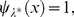
(9)is composed of the Laplace transform of Eq.(8), which is
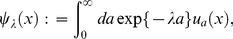
(10)and provides the fitness 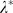 of age-size structured model [10]. According to this consequence, we then can expect to find a general age-states structured population model composed of the life history, Eqs.(1), (2), and (5). From Feynman–Kac formula (e.g., S1 in File S1), ERS satisfies the following equation:
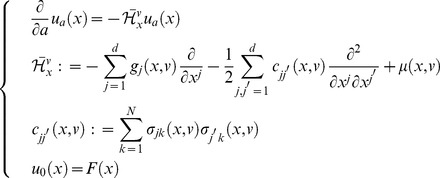
(11)Setting 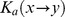 represents a transition rate (including survivorship) from initial state 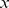 to final state 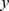 at age 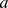 (projection function), ERS can be written by
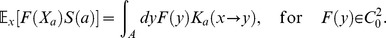
It is then known that there is a relationship between 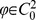 and 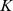 from partial integration, such that
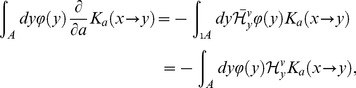
(12)where 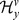 denotes the adjoint Hamiltonian of 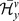 (Fokker–Planck Hamiltonian),
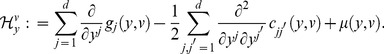
(13)From the arbitrariness of 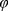, the projection function 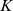 satisfies the following Fokker–Planck equation:
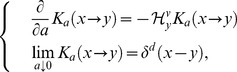
(14)where 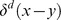 is 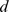-dimensional Dirac's delta
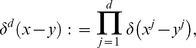
This consequence suggests that the dominant characteristic root of Eq.(10) and the projection function, 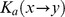, can compose the population vector, 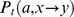, at time 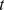 and age 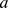 with transition from 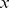 to 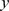 satisfying the following a stable demographic model,
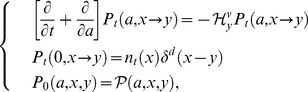
(15)because the dynamics of Eq.(11) is provided by the Fokker–Planck operator generating the cohort dynamics [10]. 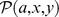 is assumed by
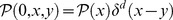
(16)The integral equation of this vector in offspring dynamics is
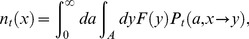
(17)where 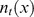 and 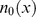 represent offspring number at time 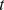 and the initial number of offspring, respectively. In comparison with Oizumi and Takada's work [10], the population vector should be decomposed into the initial offspring number and projection function, such that
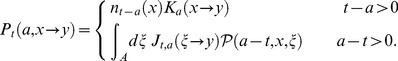
(18)In 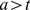, the projection fuction represents projection of states from 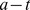 to 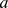 and let it satisfy 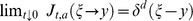. Eq.(18) is generalization of boundary condition with respect to age 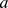 in continuous age structured models and we describe their expressions later [22]. The above decomposition can be proved in more general configuration (in non-autonomous system) of the life history by the formal solution of Eq.(15) (e.g., S1 in File S1). Setting the inner product
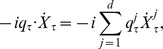
the projection function by a path-integral formalization becomes
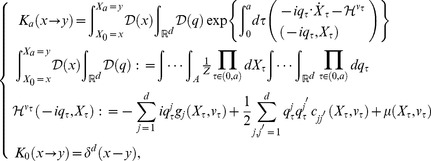
(19)where 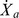, 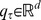, and 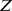 denote the formal differential of 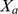 with respect to 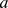, the adjoint vector, and the normalization factor of transition probability which gives
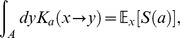
respectively. 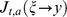 denotes the projection function in 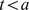 and is defined by
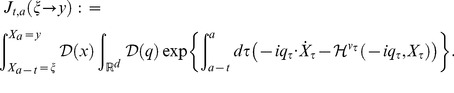
(20)When transition of all states follows autonomous system, such as Eq.(1), this function have translational symmetry with respect to age 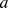 because of strong Markov property in the SDE as follows:
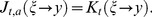
(21)The path integral is a summation over an infinity of possible growth curves connecting 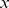 with 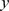 with the sieve of mortality to compute the density in Eq.(5). Eq.(19) is known in physics as the Hamiltonian expression. The algebric form of Fokker–Planck Hamiltonian, 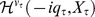, actually appears in the action integral of this expression. Substituting Eq.(18) into Eq.(17), we obtain a renewal equation of offspring dynamics as follows:
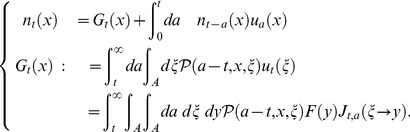
(22)Since the ERS is always nonnegative and bounded for all age 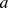 (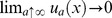) in biological assumption (Eqs.(2) and (5)), Eq.(22) corresponds with the mathematical form of the classical renewal equation which appears in McKendric equation [21]. Therefore, we can adopt Feller's methods [23] to solve Eq.(22) because this equation satisfies all conditions to use Sharp–Lotka–Feller theorem [21]. The population vector, Eq.(18), then is
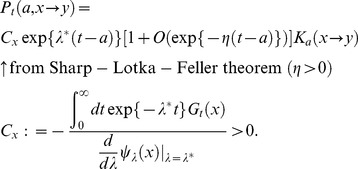
(23)Then, 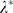 is provided by the dominant characteristic root of Eq.(9) (e.g., P.80 at [10]). Consequently, 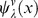 satisfies the theorem proved by Taylor 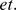 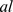 (1974) and Leon (1976) because the function is monotonically decreasing in 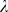 [11], [12]. The theorem shows that a strategy maximizing 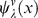 is equivalent to maximizing the fitness, 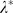. Therefore, we can adopt 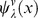 as objective function in optimal life schedule with stochastic development. Hereafter, we call 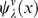 “objective function”.

#### Correspondence of path-integral formulation to other LDMs

In this part, we show the correspondence of Hamiltonian and projection function in our formalization to elements and parameters in the other formalization of LDMs: IPM, PDE, and TMM. Mathematical correspondence among those three models is introduced in some text books once in a while, such as [1], [24]. For instance, Takada and Hara provided an example that elements of a size structured TMM (Lefkovitch matrix) can generate size structured PDE (Fokker–Planck equation) at 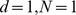 [25].

In our formalization, the projection function composing the solution of Eq.(15) can serve as the following IPM because of its Markov property [10]:
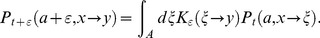
(24)On the other hand, We can show an age-size TMM corresponding to Eq.(22) at 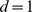 and 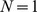. Let
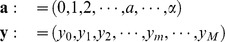
and 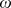 be the age vector, the size-vector, and 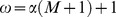, respectively. Setting a compact set 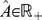 and size interval 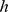 deviding 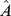 into 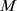 categories, 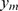 represents 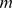-th size category. When 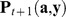 denotes a population vector in the TMM, we can show the following 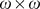 TMM:
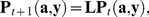
(25)where
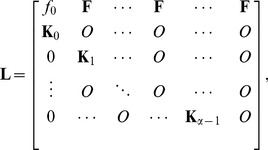
(26)is a candidate of the discretized Eq.(24) in 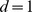 and 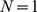. This TMM is common with Leslie matrix on the mathematical form, however, each element (vital rate) is composed of the following matrices: 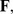 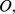 and 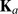 are 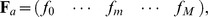 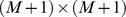 zero matrix, and 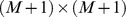 size transition matrix if and only if 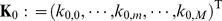, respectively.

Suppose that 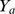 is a stochastic process of the size growth generated by the following parameterized collection of random variables,
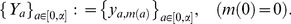
(27)The fitness follows the characteristic equation of 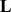 (e.g., S1 in File S1)
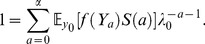
(28)This characteristic equation parallels Eq.(9) and represents the generalized Euler–Lotka equation in the TMM to age-size model. Thus, path-integral expression is hence one of formulation in LDMs. There are two reasons for adopting Hamiltonian expression of the path-integral; The first is easy to treat any cases even if 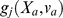 and 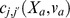 are not differentiable and including zero, such as 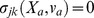, respectively and the other is of use to deal with the relationship among Hamiltonians involved in the states transition and the optimal schedule problem in the next section.

### HJB equations and analysis

We suppose
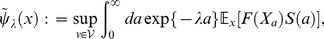
(29)from Eq.(10) in this section, because the theorem of optimal life schedule (e.g., S2 in File S1) shows that optimal strategy of life history is equivalent to finding a function 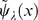. This function is called “value function” in control theory. We adapt Bellman's dynamical programming represented by PDE in stochastic control theory to find optimal control in both types of life history, and, address differences of the optimal control between semelparous and iteroparous species. This approach is not so famous method compared with “optimal life schedule problem” (OLSP) associated with “Maximum Principle (MP)”. Although there are some reasons, precursors pointed out that Bellman's approach needs the value function having sufficient smoothness to use partial differential in the analysis (e.g., Pontryagin's classical text book [26]). On the other hand, the famous approach which is MP does not need this sort of smoothness. However, the situation is changed by the appearance of viscosity solution in 1980's [27]–[29]. That is an extension of solution in differential equation and can eliminate the smoothness from the necessary condition in Bellman's approach. Moreover, it is enable for us to proof the equivalence relationship between both approaches via Hamiltonians appeared in both analyses (e.g., S3 in File S1) [30], [31]. Those two approaches have different biological points of view that Bellman's approach is to find optimal behavior as cohort dynamics represented by PDE, whereas MP is to find that as individual states transition represented by SDE. As mentioned previously, mathematicians showed that those approaches produced the same consequence without their intention. In this section, we adapt known mathematical consequenses of Bellman's dynamical programming to the value function and derive general biological interpretations from that value functions in semelparous and iteroparous species are characterized by different equations. In the next subsection, we handle the relationship between the stochastic control theory involved in dynamical programming and OLSP.

#### HJB equation and OLSP

In oreder to analyze the value function, Eq.(29) and derive the equation what it obeys, we introduce ERS with 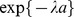


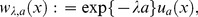
(30)and a new value function described by age backward is;
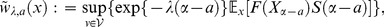
(31)to use the following relationship:
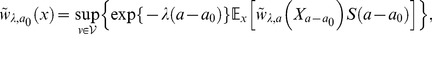
(32)where 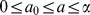. This equation implies that value function controlled from 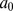 to 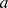 corresponds with 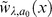 by using Eq.(31) (known as Bellman principle). Note that 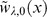 represents the value function at age 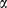 in the original variable of 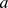. Then, Eq.(29) is rewritten as
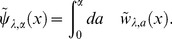
(33)Due to Eq.(9), the population growth of the fittest species asymptotically follows the fitness, 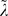, which is characteristic root of
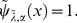
(34)Applying Feyman–Kac formula to Eq.(32), we have
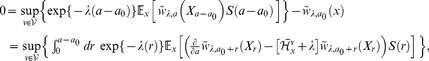
(35)Cosidering a limit
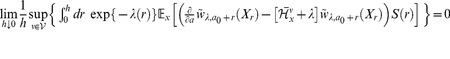
(36)where 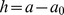, it provides an important equation (e.g., proposition and its proof on pp.182–183 in [31]) that the value function holds as follows:
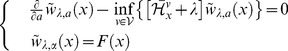
(37)This equation is known in control theory as Hamilton–Jacobi–Bellman equation (HJB equation) and essential of this study. When a control 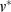 satisfies Eq.(37) or 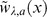 exists under the control, 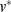 is optimal [14], [32]. Eq.(37) is, however, nonlinear and 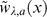 generally does not have sufficient smoothness; the value function is interpreted as a “viscosity solution” in Eq.(37) (e.g., S4 in File S1 and [30], [31]).

Since 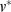 has a degree of freedom with respect to 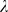 and has to hold Eq.(34), the unique optimal control is given by 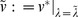. Those two procedures are biologically essential because the optimal control depending on 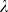 means optimizing not only the ERS but also the speed of alternation of generations. In other words, to maximize the objective function is to maximize the ERS of precocious individuals of which has small reduction by 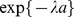. Since 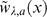 depends on age and states, it suggests that an optimal control, 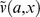, also depends on both state 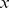 and age 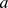. Therefore, species having the optimal state transition at the original age, 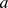, (age forward) becomes
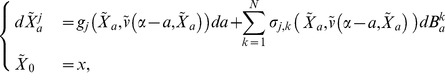
(38)from Eq.(1). Due to Eq.(38) being non-autonomous system, the projection function requires more general form than Eq.(19) as follows:
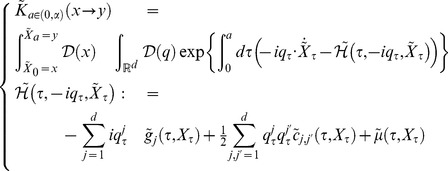
(39)where
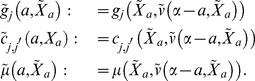
(40)This path-integral formulation admits a case that terms, 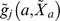, 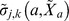, and 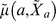 are not always differentiable everywhere.

#### Stationary control problem of life history

If species die only by accidental death or programed death after their reproduction (such as semelparous life history or life history having sufficiently long lifespan), the case can adapt to their analysis, we can deal with the optimal control in a case 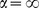, i.e.,
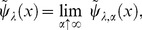
(41)from Eq.(33). Since 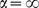 contain all mature ages individually fluctuated by internal stochasticity, we adopt this assumption to analysis of semelparous species in the next subsection. Additionally, it is known that the value function, Eq.(41), is the solution (in the viscosity sense) of another HJB equation:
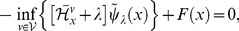
(42)
[32]. Analysis of the optimal control which satisfies this equation is known as “stationary control problem” in control theory. This control is essentially distinct from one satisfied Eq.(37), because Eq.(42) does not depend on age. Consequently, the optimal state transition becomes the autonomous system as follows:
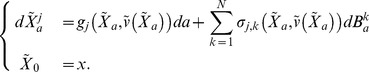
(43)As Eq.(42) directly provides the value function, this case requires only two equations: Eqs.(34) and (42).

#### Optimal life histories and HJB equations in semelparous and iteroparous species

As mentioned above, semelparous optimal control is categorized as stationary control problem on account of the random mature age. Due to the strong Markov property of the SDE, Eq.(43), the objective function of semelparous species, 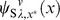, can be generally written as
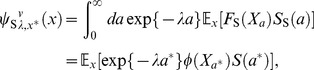
(44)we can simplify this equation if and only if 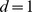 and the boudary is one point (e.g., S4 in File S1). Then, semelparous species have two optimal life schedule problems: (1) how to determine the optimal mature state and (2) how to control the growth rate until that states are reached. The former is known as the “Optimal stopping problem” in probability theory, as mentioned in [10]. To simplify the latter problem, we assume in this subsection that the optimal mature state exists and given. Setting the optimal mature age, 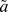, a value function of semelparity becomes
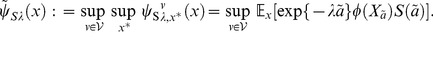
(45)This equation suggests that the optimal control optimizes the expectation of survivorship until the mature age, 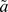. In other words, the evolution of a semelparous life history implies the optimization of life span.

From Eqs.(4) and (42), the semelparous value function satisfies the following Dirichlet boundary value problem of HJB equation:
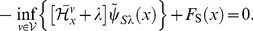
(46)When Eq.(46) has a unique solution in the viscosity sense, we can obtain an optimal control 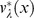, such that
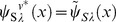
(47)from the theorem on p.228 in [31]. From the semelparous fertility function, Eq.(4), the value function satisfies
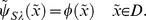
Therefore, the optimal control of semelparous species, 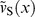, (a Markovian control) becomes
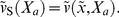
(48)As shown in the previous subsection, the optimal cotrol does not depend on the age.

On the other hand, iteroparous species reproduce during their growth: the value function becomes
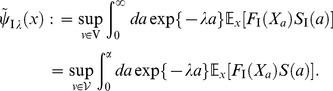
(49)Therefore, the iteroparous value function is given by the solution in the viscosity sense of Eq.(37) and the characteristic equation Eq.(34). In other words, the evolution of iteroparous species implies the temporary optimization of the ERS which is different from the semelparous breeding system. Moreover, the maximum age 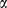, provides a terminal condition to the ERS, which introduces complexity to the analysis of iteroparous species.

#### Density of breeding age structure in fittest

We demonstrate, here, that this unification theory of OLSP and LDM can address the statistical characteristics of population structure associated with the optimal life history. We focus on statistics of breeding age; Oizumi and Takada showed objective function directly implied cumulant generating function (CGF) of that [10]. We then extend their consequence to our configuration. Let 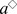 be breeding age. The CGF is written as a Taylor series of the logarithm of objective function with respect to 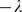 as follows:
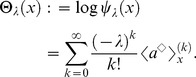
(50)Probability density 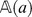, generating the CGF, is composed of basic reproductive number (known as 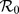) and ERS by using Eq.(10) such that
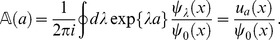
(51)If we can obtain the fittest 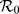, ERS, and cumulant generating function of breeding age directly from the value function, as for an objective function, analyzing the fittest population structure is useful. When one applies Eq.(29) to the cumulant generating function as an objective function in the optimal control, Eq.(29) should be a Laplace transform of the ERS. This means that the optimal control should not be a function of 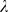 because expanding the logarithm of Eq.(29) into a Taylor series as Eq.(50) causes a loss of the meaning of the objective function in the optimal control. This holds true as long as maximizing the ERS is equivalent to maximizing fitness. The key point to calculate in the case is the second term of the RHS of another expression of the value function, Eq.(31):
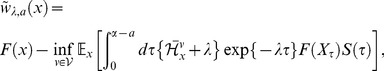
(52)obtained by integrating both sides of Eq.(37) for age 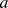 (Dynkin's formula [31]). In the iteroparous breeding system, we can show a condition that the value function can compose statistics of breeding age. Differentiating the integrant of the second term in Eq.(52) with respect to 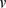, we obtain the condition that 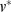 satisfies;
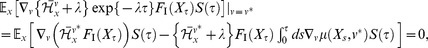
(53)where 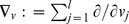 and 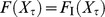. This equation illustrates that the optimal control does not depend on 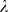 if and only if the mortality does not include the control vector. In other words, deriving the optimal control maximizing the ERS from Eq.(52) without calculating the Euler–Lotka equation directly is sufficient in this case. From a biological point of view, individuals of the species do not control their life spans in this case: mortality is not susceptible to mutation or strategy change, or is already optimized. In general, an optimal control provides the adaptive ERS and life span because altricity and precocity increase the fitness in 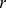-selection [33], [34]. In other words, 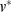 being unconnected to 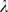 means excluding the control of precocity. According to the analysis of optimal control in semelparous breeding timing [10], precocity serves as a positive influence on persistence of the species under internal stochasticity. This consequence was explained by the following reason: the internal stochasticity yields individuals having precocity and late-maturing. The former individuals accelerate alternation of generation, namely, they contribute to increase their fitness. Since the others are exposed by higher risk of death, their contribution affects on the fitness and the 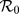 negatively. Proportion of both individuals is given by their maturity, such as mature body size and fertility, and affects on their persistence. In contrast, empirical researchers reported that short-lived species had vulnerability for external stochasticity by sensitivity analysis in a geometric mean of each dominant eigenvalue of interannual TMMs (long-term population growth rate) [8], [35]. When species has control strategies excluding that of mortality, long-lived iteroparous species (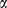 is large) should be adaptive because 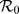 is monotonically increasing in 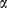. This consequence seems to consist with those reports. However, the other cases remain a distinct difference between the effect of internal and external stochasticities because the short-lived species can be adaptive under the former. Although this paper handles only internal stochasticity, there may exist a trade-off between both adaptations for stochasticities for evolution of long-lived and iteroparous species. When mortality is not controlled, the value functions can directly compose the cumulant generating function of breeding age 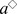 in the fittest population structure such that
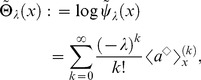
(54)and
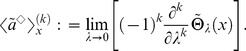
(55)By using the original age, the density of 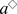 in the fittest population structure is given by the following function:
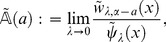
(56)where 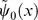 means 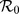.

In the next section, we examine several specific models in semelparous and iteroparous breeding systems and discuss their differences.

### Applications

We show analysis of specific models in this section. Oizumi and Takada (2013) showed that internal stochasticity causes both increase and decrease of fitness [10]. Focusing on the latter case, the stochasticity is a kind of risk for species. We suppose that species have a choice to utilize two resources. Traits of these resources are different from each other, such that one of these gives the effect of “high risk and high return” and that the other gives the effect of “low risk and low return” on the individual size growth. As application of our theory, considering the optimal utilization of those resources, we show the effect of the utilization on the fitness. Accordingly, the species suitably averting risk maximizes the fitness in their habitat.

#### Two-resource utilization model

**Figure 1:**
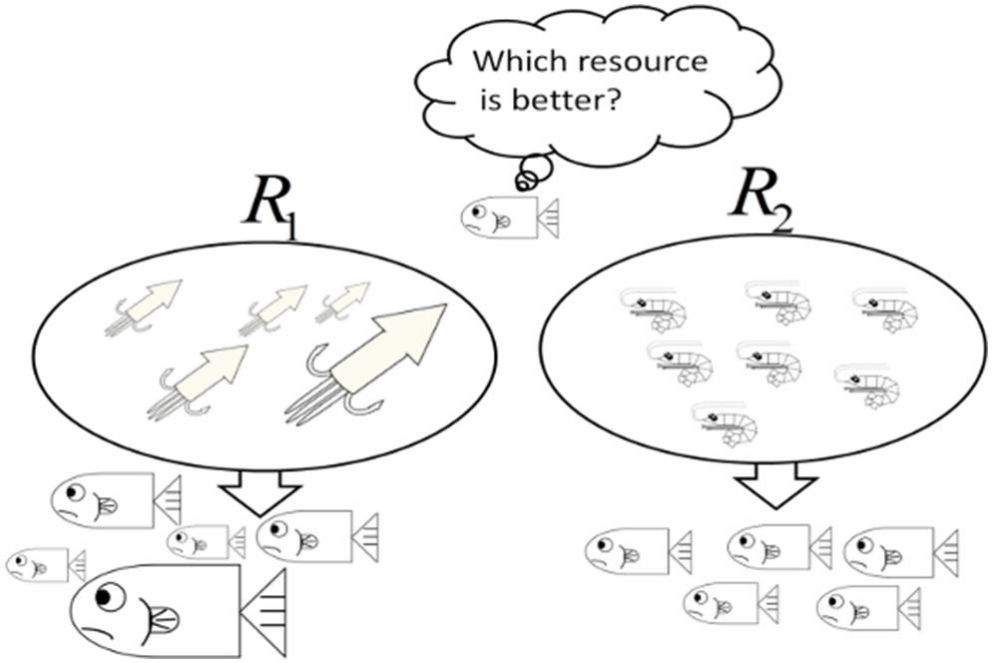
A resource utilization problem. This figure illustrates how a fish wavers in its choice. 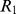 represents high risk but high expected growth rate, while both quantities are low in 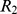. These resources fluctuate independently of each other. In other words, the two resources provide individuals with different internal stochasticities. The fish should optimally choose the best utilization of both resources.

We consider a simple size growth model as an application of our theory. When 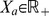 is a body size at age 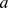, we assume that the species can choose two kinds of resources: the species using a resouce, 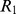, have the following growth rate of size such that
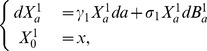
(57)and another resouce, 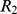, also being
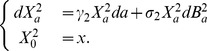
(58)Thus, we assume that resources 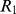 and 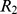 operate life histories of individuals via their growth rate of size. Let 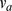 be utilization frequency which represents a frequency of utilization in both resources. The individual growth rate of size, then, follows
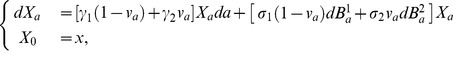
(59)We assume 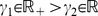 (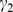 could be negative), 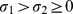 i.e., 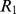
–specialists (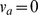) have higher risk and the expectation of growth rate than 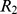
–specialists(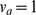). Conversely, 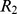
–specialist has the other risk that the individuals have lower survivorship until mature age than 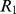
–specialists because of slower growth rate on the average. Therefore, individuals should find an optimal “utilization frequency” 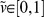 to minimize the Hamiltonian with 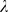 under the environment (e.g., Fig. 1).

Eq.(59) is well-known as typical “optimal portfolio selection problem” in mathematical finance and economics. Economists apply the problem to find an optimal investment of their wealth [13].

We assume a mortality in Eq.(5) such that
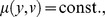
where we set
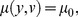
(60)which does not have control. We are interested in difference of the optimal utilization 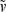 of Eq.(59) in different breeding systems. As preparation for analyzing the optimal utilization, the adjoint Hamiltonian of Eqs.(59) and (60) becomes
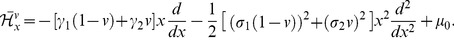
(61)Let 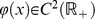 provide an extreme value of 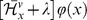 with respect to 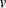 such that
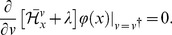
(62)Then, the value satisfies
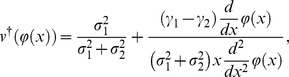
(63)and we obtain a nonlinear operator by substituting Eqs.(63) into (61) as follows:
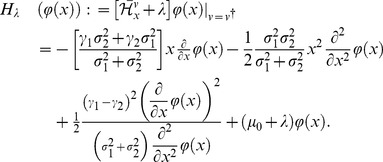
(64)Hereafter, we use frequently those equations, Eqs.(63) and (64), in our analysis.

#### Reproductive timing in semelparous species

This subsection describes results optimal reproductive timing in [10]. They used a case that Eqs.(57), (4) and (60) (or (58)). The objective function 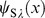 then staisfies
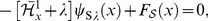
(65)where 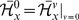. The solution of this is
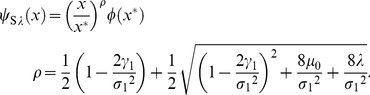
(66)Analysis of the above objective function showed that the optimal mature body-size 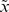 satisfies
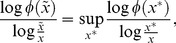
(67)It is convenient for analyzing Eq.(59) that the optimal mature age does not depend on parameters such as, 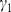, 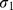, and 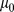. Therefore, the model has the same optimal body-size and age irrespective of the control parameter, 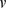. Setting
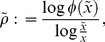
(68)they showed the fitness by using 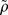 as follows:
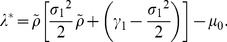
(69)It was shown that the stochasticity decreases the fitness if and only if 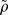 is less than one. In other words, the optimal utilization possibly exists at 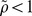 in the model. If 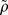 is more than one, 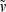 of Eq.(59) is obviously equal to zero because the stochasticity affects the fitness positively. In persistent species, large 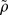 makes species advantageously at any parameters.

## Results

### Semelparous optimal resource utilization

From Eq.(46), the value function satisfying Eqs.(4), (59), and (60) is generated by the following HJB equation:
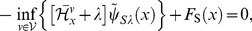
(70)where 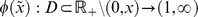 in this case. Since Eq.(70) is a quadratic function of 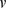 (e.g., Eq.(61)), the extreme value is determined by 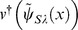 from Eq.(63). Substituting the value into Eq.(70), we obtain a nonlinear ODE from Eq.(64) as follows:
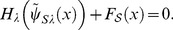
(71)To find the solution of Eq.(71), we assume a solution 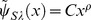 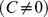 at 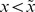, and substitute it into Eq.(71) as follows:
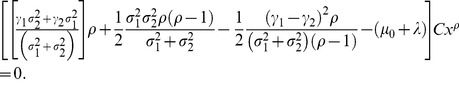
(72)Setting 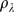 to satisfy Eq.(72), we obtain
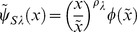
(73)and the constant, 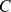, is given by
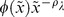
to satisfy the boundary condition in Eq.(46), i.e., 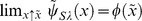. Eq.(73) can provide an unique optimal frequency 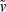 (e.g., the theorem on pp.228–232 in [32]) despite that 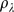 possibly have three different values, because the characteristic equation Eq.(9):
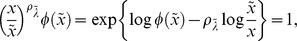
gives the following relationship
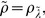
(74)from Eq.(68). Furthermore, Eqs.(72) and (74) can provide a unique fitness to be mentioned later. If 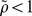, we can find the optimal control from Eq.(63) because the value function, 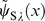, becomes a concave function.

**Figure 2:**
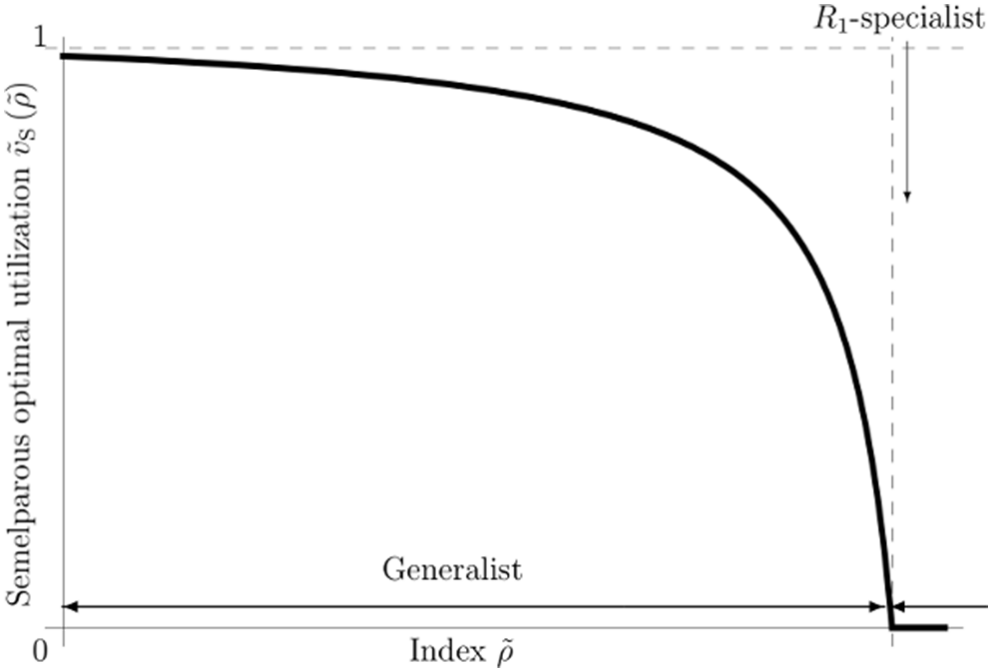
Semelparous optimal utilization for each 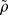. This figure shows the semelparous optimal resource utilization, Eq.(76), depending on 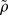. Two distinct types of feeding habitat exist. A small or intermediate value of 
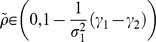
 makes the species a generalist: the larger the value of 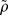, the more the species favors risk and becomes specialist. Parameters are 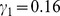, 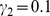, 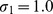, 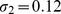, and 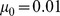.

Substituting Eqs.(73) and (74) into Eq.(63), the optimal utilization, 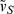, becomes
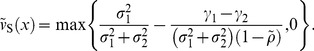
(75)This optimal utilization is constant, and it means that the species should conserve a frequency of resources during its lifetime. Regarding 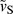 as a function of 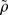, 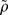 is an important index to determine the utilization. If the index is large, it shows that the semelparous species has a tendency toward risk appetite (e.g., Fig. 2) because 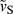 is small. Then, the optimal resource utilization continuously changes with respect to 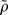. Since the continuity of optimal control with respect to 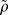 is provided by the second-order term of 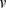 in the diffusion term of the Hamiltonian, Eq.(61), it is different from the bang–bang controls appearing in deterministic models, which do not have second-order terms of control parameters.

**Figure 3:**
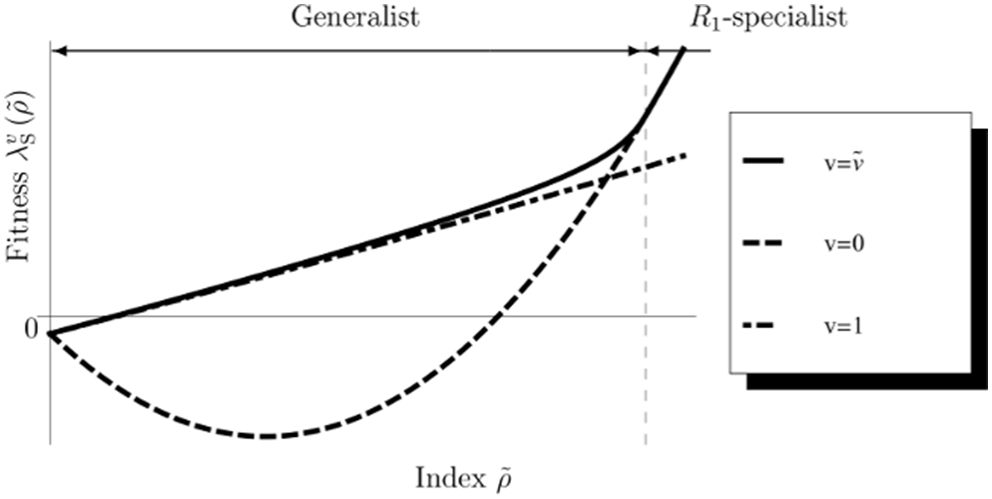
Fitness of optimal utilization in semelparous species. We calculate the value of the fitness of semelparous species with respect to 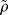 between zero and 1. The vertical dashed line represents the boundary between specialists and generalists as given by Eq.(77). The fitness is always a monotonically increasing function of 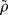 in the persistent region of the species (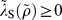). It is remarkable that a large 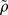 causes individuals to favor risk and increase their fitness. This figure shows the optimal resource utilization actually having advantages over specialists of each of the resources. Parameters are the same as in Fig. 2.

From Eqs.(69), (72), and (74), we obtain the fitness of the fittest as follows:
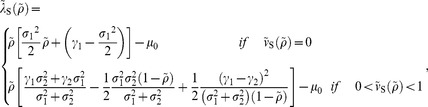
(76)from the RHS of Eq.(72) (e.g., Fig. 3). If 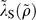 is non-negative, the species is persistent. In that case, the fittest becomes 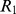-specialist or generalist and 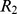-specialist is never selected because of magnitude correlation of each parameter in our definition. Nature selects generalist when 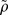 is within
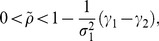
(77)otherwise 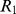-specialist is selected.

**Figure 4:**
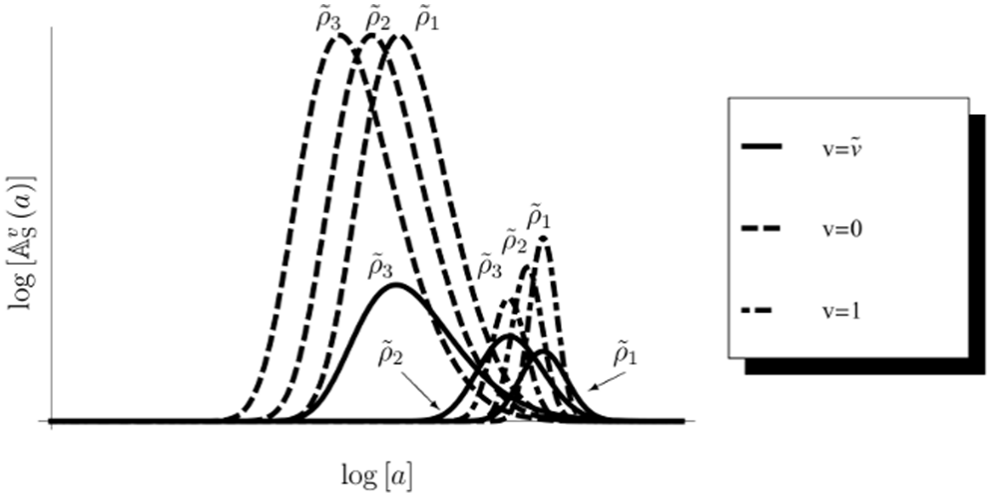
Transition of the mature age distribution. Whenever 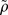 becomes large (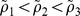), the mature age distributions of every utilization behavior are shifted to younger ages than those under smaller 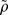 as in [10]. If the fittest species is a generalist, the change in distribution becomes extreme. Then, the age distribution is provided by S5 in File S1. We chose 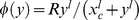 as the fertility rate function. This function has a unique 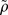 with respect to 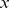, and the optimal body size, 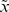, is obtained by calculating the derivative of 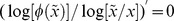 with respect to 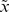. We substitute the RHS of that equality into 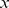 of the distribution. Then, we obtain this figure by changing 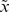. Then, 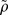 becomes inversely proportional to 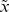 in this fertility function. Parameters are 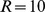, 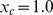, 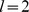, 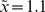, 1.3, 1.5, with the others being the same as in Fig. 3.

**Figure 5:**
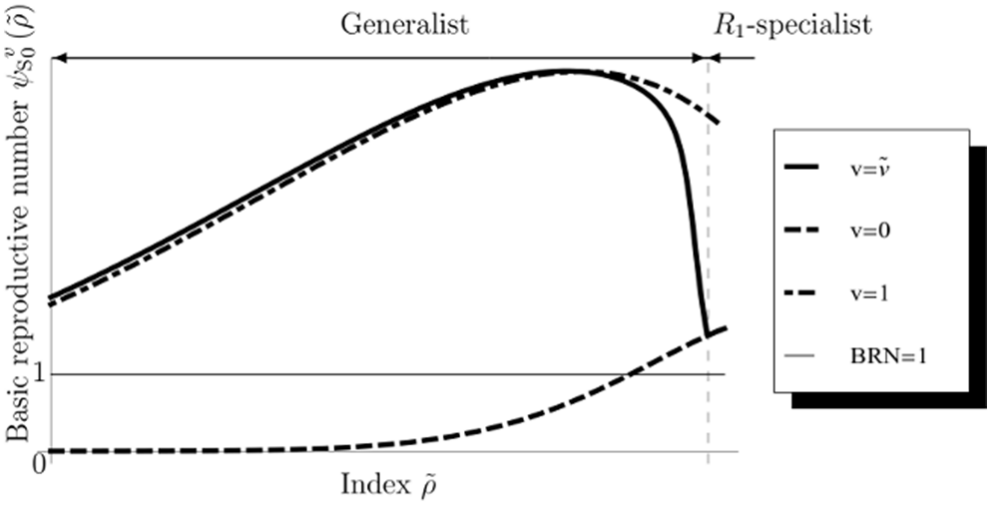
Transition of the 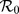. This figure shows changes in the 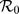 with respect to 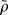. The 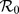 of the fittest species increases as 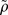 increases; however, it decreases when 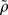 reaches the RHS of Eq.(77), which means that the growth strategy changes from the conservation of the 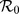 to a hasty alternation of generation time. Utilizing 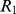 usually, the 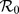 of the fittest is higher than that of 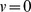. We simulate the 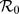 under the same parameters as in Fig. 4. To show the proportional connection between 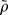 and the 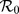, we change the variable (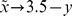) and simulate it within (0, 3.5) because 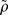 is in inverse proportion to 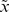. Parameters are the same as in Fig. 4.

The dependence of utilization on 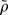 can be explained by a trade-off between the alternation of generation and 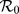. It was shown in [10] that the internal stochasticity increases the number of precocious individuals, however, decreases that of mature individuals. A large value of 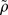 expresses a small difference between a mature body size and the initial body size, a large fertility 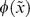 or both, and makes the reproduction number of precocious individuals compensated for the decrease of number of mature individuals. Small 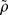 makes the opposite consequence that the reduction of the number of mature individuals exceeds the reproduction number of precocious individuals (e.g., Fig. 4 and Fig. 5).

### Iteroparous optimal utilization

In this subsection, changing from the breeding system of Eq.(4) to 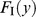, we compare the optimal strategy of semelparity with that of iteroparity. We use the value function from Eqs.(31) and (49), and the same Hamiltonian as in Eq.(61).

We assume the fertility function to scale with body-size allometric law, such as for the biomass of shoots and body size in trees [36], as follows:
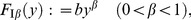
(78)where 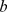 and 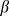 represent the fertility rate and an allometric exponent within the domain 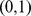, respectively. Using the Hamiltonian, Eq.(61), we can obtain an optimal utilization of the iteroparous species identical to the one of the semelparous species. From Eqs.(37) and (63), the value function in Eq.(31), becomes a solution of the nonlinear PDE
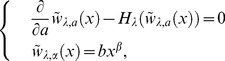
(79)because
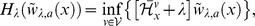
and the optimal utilization satisfies
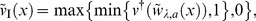
(80)from Eq.(63). To derive the solution of Eq.(79), we assume
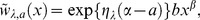
(81)and substitute it into Eq.(79). Then, 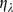 becomes
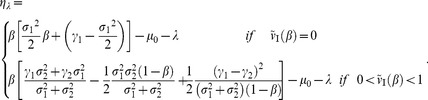
(82)Eq.(81) is guaranteed as an unique solution of Eq.(79) from the uniqueness of the viscosity solution [28]. Namely, 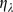 can be expressed by using the function of semelparous fitness, Eq.(76), such that
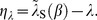
(83)This suggests that the iteroparous optimal utilization has common and different characteristics compared to the semelparous one. We here use the expression, Eq.(83), to emphasize the common characteristics between semelparous and iteroparous optimal life histories. Substituting Eqs.(79) and (82) into Eq.(80), we obtain the optimal utilization as follows:
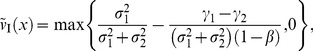
(84)without having to solve the Euler–Lotka equation, as the mortality does not include control parameters. When we regard the utilization, Eq.(84), as a function of 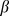, the functional form resembles the optimal utilization of semelparous species, (Eq.(76)). Then, the exponent, 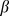, serves as 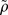 in semelparous species.

Using Eqs.(49), (81) and (82), the iteroparous value function in Eq.(49) becomes
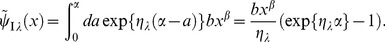
(85)Since Euler–Lotka equation, Eq.(9) being composed of Eq.(85) becomes transcendental, we cannot find the fitness of the iteroparous species explicitly. Then, we use the following inequality
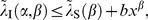
(86)where the RHS of Eq.(86) is the dominant characteristic root of
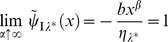
from the Euler–Lotka equation, Eq.(9), applying the “Basic optimal life schedule theorem” (S2 in File S1) to 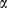. Since individuals in initial states normally contribute little to reproduction in nature, we roughly assume that 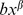 is sufficiently small.

Considering the persistence of the species (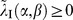), 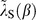 should be non-negative. In this model, the optimal utilization is supposed to maximize not only the fitness but also the ERS of the original age:
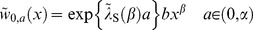
(87)Additionally, if the fitness, 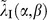, is a monotonically increasing function in 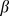, 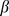 has an identical meaning to that of 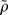 in semelparous species. To prove this, we show that the ERS increases monotonically in 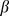. Since 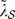 satisfies
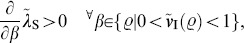
and Eq.(69) is a monotonically increasing function of 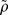 in 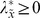, we conclude the proof.

From Eqs.(56), (87), and the 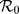:
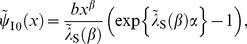
(88)the breeding age density becomes
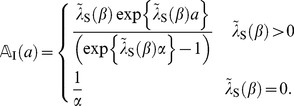
(89)If 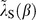 is positive, the age density shows that it skews toward older ages. In this case, the contribution of older individuals to reproduction is important for the persistence of the species. Even if 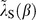 is equal to zero, the contribution is not negligible because the density has a uniform distribution. Since Eq.(76) maximizes the fitness of semelparous species, 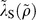, the optimal utilization of iteroparous species also maximizes 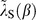. Although for large values of 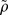 and 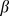 species in both breeding types favor more risky behavior, their breeding age structures are different, such that the semelparous mature age density is L-shaped and that the other is J-shaped (e.g., Fig. 4 and Eq.(89)). Consequently, the optimal utilization of iteroparous species enhances the contribution of older individuals to reproduction and differs from that of semelparous species because their longevity provides them with many opportunities for reproduction and with sufficient time to reach a large size.

Considering the persistence of the species (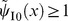) with respect to the maximum age, 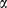, the following relation should be satisfied
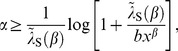
(90)from Eq.(88). Since Eq.(85) increases monotonically in 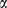 and Eq.(86) is proved by using the basic optimal life schedule theorem, iteroparous fitness is a monotonically increasing function in 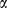. Therefore, iteroparous species evolve to have optimal utilization and to survive as long as possible, as found for trees.

## Discussion

This theory generalizes from the previous study [10] and extends to general OLSP in LDMs. The path-integral formulation is more suited to address controlled life history than the others (PDE, TMM, and IPM) because it conserves continuity of states and does not require differentiablity of parameters: 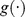 and 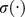. These parameters represent statistics of stage transition rate in TMM; they appear as components of Hamiltonian in the path-integral formulation. This Hamiltonian has a significant meaning in OLSP because the optimal control of life history minimizes or maximizes it depending on fertility function. There are two approaches in control theory that are to solve the HJB equation and maximum principle (MP). The analysis of optimal stochastic control using the HJB equation has been developed in various fields, including engineering and finance. However, many theoretical biologists commonly have used the MP approach in the analysis of OLSP [15], [16], [37,37]. The stochastic MP is proved by Peng (1990) [39], but it is not overwhelmingly popular in theoretical biology. According to Yong and Zhou (1999) [31], both methods are formalized by the common Hamiltonian. There are two different viewpoints in those approaches. Idea of MP is originated from Hamilton's canonical system which is based on dynamics of a particle system in classical mechanics; i.e., OLSP based on MP describes an individual optimal life history. On the other hand, the idea of HJB equation is originated from Hamilton–Jacobi equation (PDE); it describes the optimal life history as optimizing cohort dynamics. These two approaches are equivalent in the sense that solutions of one can be represented by those of the other. Additionally, the correspondence of the HJB equation to the stochastic MP was shown via the idea of a viscosity solution by precursors [28]–[31], [40]. They showed that the value function and its derivatives in the HJB equation correspond to the co-state variables in the stochastic MP. The Hamiltonian, which mathematical biologists use in the OLSP associated with MP approach, was merely one of the mathematical procedures for the analysis. The Hamiltonian now forms an important element of the demographic model. The path-integral formulation unifies stochastic control theory and LDMs via the Hamiltonian, and we showed that optimal strategies usually minimize 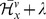. This Hamiltonian, 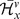, forms a counterpart of the Fokker–Planck Hamiltonian, referred to the “adjoint Hamiltonian” in Eq.(11). In physics, the Hamiltonian refers to the total energy of the system. Using this analogy, we can interpret Hamiltonian as a biological energy, whereby individuals consume energy throughout their lifetime. Then, the value function derived from the HJB equation represents the lowest energy consumption over the lifetime, and the Euler–Lotka equation converts the life history into population dynamics. Therefore, we can omit the analysis of population dynamics in the LDM because the theory in this study showed that the following equations provide the unification of OLSP and LDM:
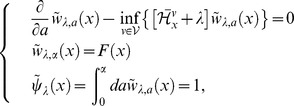
or another HJB equation:
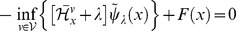
if and only if the species does not have a terminal condition, such as maximum age, from Eq.(37).

The two-resource utilization model shows that optimal strategies behave differently depending on breeding systems and fertility functions, even if a species occurs in the same habitat. Then, the convexity of the objective function is the keyword of all of the optimal strategies analyses in this study. To explain the importance of the convexity, we introduce Jensen's inequality. Let 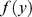 be a concave function with respect to 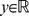, such as 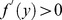 and 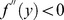. Then, the function satisfies the following inequality for an arbitrary random variable 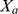,
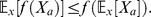
Incidentally, as for the convex function, the inequality becomes an opposite magnitude correlation. As Jensen's inequality suggests, the exposure of a species to risk becomes advantageous when the objective function has high convexity. Since the convexity depends on life history, semelparous and iteroparous species possibly have different growth strategies even if they share several elements of life history, such as Eq.(1), and 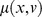 in the habitat. In this case, the difference in fertility yields different convexities of the objective function and strategies. In other words, the breeding system determines what the species optimize.

In semelparous species, the parameter, 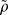, represents an index of convexity degree in the value function. Although the previous study [10] did not mention the convexity, the positive effect of internal stochasticity is caused by the strength of the index. As Oizumi and Takada (2013) reported, there was a trade-off between precocity and altricity in the OLSP. The stochasticity yields both precocious and slow-growing individuals. The former makes alternation of generations faster, while the latter decreases 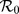 by increasing the risk of death. The paper showed that the index, 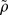, determines the sensitivity of 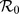 in the stochasticity. Especially, a small 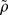 decreases 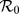 in the domain, 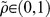. Consequently, species having a small 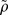 utilizes smaller risk, such as 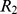. The optimal utilization, 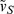, continuously decreases with 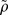. Then, nature selects a species of specialists or of generalists in resources depending on 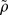.

The risk appetite of semelparous species depends on 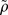, composed of the mature body size and 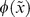 in the optimal utilization, while that of iteroparous species depends on the allometric exponent 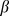. This has the same characteristic as the index 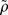 in the optimal utilization of semelparous species. If a species has a large value of 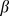, the fitness is also high. Those parameters characterize the convexity of the objective function. As Jensen's inequality shows, the convexity determines the effect of internal stochasticity on fitness. A large value of 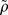 or 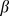 makes the objective function close to a convex function. When they have the same convexity, 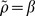, semelparous and iteroparous species have the same risk appetite and the optimal utilization.

In contrast, the breeding age distribution of iteroparous species is different from that of semelparous species. The trend in age distribution depends on 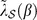. If 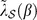 is positive, the breeding age distribution skews toward older ages (e.g., Eq.(89)). Then, the persistence of the species is determined by the maximum age 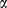. Therefore, long-lived individuals are important for population growth in this case.

Another meaning of 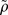 and 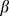, from a biological point of view, represents the efficiency of conversion from adult body size to number of offspring. For example, a large value of 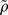 means a mature individual producing many offspring and/or having a low ratio of mature body size to initial body size. Therefore, the risk appetite is determined by the efficiency of conversion. The evolution of generalists is considered to be related to a portfolio effect in our resource utilization model. The portfolio effect is a species diversifying its resource utilization and diet to reduce risk [41]. This effect has been reported in various cases, such as in ascent of salmons [42]. This study shows that the diversification of resource is important for species susceptible to internal stochasticity (i.e., when 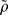 and 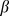 are small), which may provide an explanation for the evolution of the portfolio effect.

Our application shows several kinds of optimal growth strategies depending on breeding systems in a simple stochastic growth process. However, our theory (Eqs.(9), (10), (37), and (46)) is applicable to other events and trade-offs in the life histories of organisms. The controls maximizing the objective function and 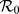 have basically different meanings. The control of objective function simultaneously optimizes the ERS and generation time. In contrast, the latter control of 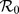 maximizes only the 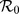. Therefore, the optimal control that maximizes the objective function is more complicated control than the latter. Conversely, Eq.(53) shows that both types of controls accord in iteroparous species when the mortality does not depend on the control parameter. The analysis of iteroparous species does not need to consider the effects of control on generation time because the model satisfies the condition mentioned above. Considering the mortality controlled, another trade-off occurs between the risk of stochasticity and survivorship. The methods presented in this study are suitable for use in addressing such issue and provide a basis for such future research.

## Supporting Information

File S1
**Supporting material.** S1:Analysis involved in path-integral. S2:Basic theorem of life strategy. S3:Viscosity solution. S4:Derivation of a general 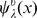 in semelparous species. S5:Mature age density of semelparous species.(PDF)
