# Supplementary material for: Unification Theory of Optimal Life Histories and Linear Demographic Models in Internal Stochasticity
Source: PLoS One. 2014 Jun 19;9(6):e98746. doi: 10.1371/journal.pone.0098746 (PMC4063715; doi:10.1371/journal.pone.0098746)
Supplement: File S1 — Supporting material. S1:Analysis involved in path-integral. S2:Basic theorem of life strategy. S3:Viscosity solution. S4:Derivation of a general in semelparous species. S5:Mature age density of semelparous species. (PDF) [file pone.0098746.s001.pdf]

Supporting infomation

S1

Feynman–Kac formula

Feynman–Kac formula is usefully to analyze dynamics of diffusion process in a potential medium. In a sense, the formula represents a differentiation formula which is extended to stochastic differential such that

$$d\left(\varphi\left(X_a\right)S\left(a\right)\right) = d\varphi\left(X_a\right)S\left(a\right) + \varphi\left(X_a\right)dS\left(a\right), \tag{S.1}$$

where $\varphi \in C^{\infty}\left(A\right)$ Then, it becomes

$$\begin{aligned} d\left(\varphi\left(X_a\right)S\left(a\right)\right) = &\left[\sum_{j=1}^{d} g_j\left(X_a, v_a\right)\frac{\partial}{\partial x^j} + \frac{1}{2}\sum_{j,j'=1}^{d} c_{jj'}\left(X_a, v_a\right)\frac{\partial^2}{\partial x^j \partial x^{j'}}\right]\varphi\left(X_a\right)S\left(a\right)da \\ &- \mu\left(X_a, v_a\right)\varphi\left(X_a\right)S\left(a\right)da + \sum_{j=1}^{d}\sum_{k=1}^{N}\sigma_{jk}\left(X_a, v_a\right)\left(\frac{\partial}{\partial x^j}\varphi\left(X_a\right)\right)S\left(a\right)dB_a^k, \end{aligned} \tag{S.2}$$

from a property of stochastic differential. The Feynman–Kac formula asserted that the expectation of Eq.(S.2) holds

$$d\mathbb{E}_x\left[\varphi\left(X_a\right)S\left(a\right)\right] = -\bar{\mathcal{H}}_x^v \mathbb{E}_x\left[\varphi\left(X_a\right)S\left(a\right)\right]da.$$

Therefore, let $u_a\left(x\right) := \mathbb{E}_x^v\left[\varphi\left(X_a\right)S\left(a\right)\right]$ and we have

$$\begin{cases} \dfrac{\partial}{\partial a}u_a\left(x\right) = -\bar{\mathcal{H}}_x^v u_a\left(x\right) \\ u_0\left(x\right) = \varphi\left(x\right). \end{cases} \tag{S.3}$$

Additionally, this formula can extend to Dirichlet's boundary problem, such as $F_{\mathrm{S}}\left(x\right)$, in the distribution sense. The proof of the formula is in [1, 2].

Derivation of path-integral expression

Assuming $\tilde{g}$, $\tilde{\sigma}$, and $\tilde{\mu} \in C^{\infty,\infty}\left((0,a)\times A\right)$, we consider a non-autonomous age/states structured PDE

$$\begin{cases} \left[\dfrac{\partial}{\partial t} + \dfrac{\partial}{\partial a}\right]P_t\left(a, x\to y\right) = -\mathcal{H}_{a,y}P_t\left(a, x\to y\right) \\ \mathcal{H}_{a,y} := \displaystyle\sum_{j=1}^{d}\frac{\partial}{\partial y^j}\tilde{g}_j\left(a,y\right) - \frac{1}{2}\sum_{j,j'=1}^{d}\sum_{k=1}^{N}\frac{\partial^2}{\partial y^j \partial y^{j'}}\tilde{\sigma}_{j,k}\left(a,y\right)\tilde{\sigma}_{j',k}\left(a,y\right) + \tilde{\mu}\left(a,y\right) \\ P_t\left(0,y\right) = n_t\left(x\right)\delta^d\left(x-y\right). \end{cases} \tag{S.4}$$

Let $\left(t,a\right)$ be fixed, and ε be a new variable. The LHS of Eq.(S.4) becomes

$$\frac{\partial}{\partial \varepsilon}\bar{P}_{\varepsilon}\left(x\to y\right) = \frac{\partial}{\partial t}P_{t+\varepsilon}\left(a+\varepsilon, x\to y\right) + \frac{\partial}{\partial a}P_{t+\varepsilon}\left(a+\varepsilon, x\to y\right),$$

and we define

$$\begin{aligned} \bar{P}_{\varepsilon}\left(x\to y\right) &:= P_{t+\varepsilon}\left(a+\varepsilon, x\to y\right) \\ \bar{g}_j\left(\varepsilon, y\right) &:= \tilde{g}_j\left(a+\varepsilon, y\right) \\ \bar{c}_{j,j'}\left(\varepsilon, y\right) &:= \sum_{k=1}^{N}\tilde{\sigma}_{jk}\left(a+\varepsilon, y\right)\tilde{\sigma}_{j'k}\left(a+\varepsilon, y\right) \\ \bar{\mu}\left(\varepsilon, y\right) &:= \tilde{\mu}\left(a+\varepsilon, y\right). \end{aligned} \tag{S.5}$$

Then, we obtain a Fokker–Planck equation with respect to the new population density, $\bar{P}_\varepsilon(x \to y)$, is given as follows:

$$\begin{cases} \dfrac{\partial}{\partial \varepsilon}\bar{P}_\varepsilon(x \to y) = -\mathcal{H}'_{\varepsilon,y}\bar{P}_\varepsilon(x \to y) \\ \mathcal{H}'_{\varepsilon,y} := \displaystyle\sum_{j=1}^{d}\frac{\partial}{\partial y^j}\bar{g}_j(\varepsilon,y) - \frac{1}{2}\sum_{j,j'=1}^{d}\frac{\partial^2}{\partial y^j \partial y^{j'}}\bar{c}_{j,j'}(\varepsilon,y) + \bar{\mu}(\varepsilon,y) \\ \bar{P}_0(x \to y) = n_t(x)\,\delta^d(x-y)\,. \end{cases} \tag{S.6}$$

To derive path integral from Eq.(S.6), one uses Fourier transform of the function $\bar{P}_\varepsilon(x \to y)$ with respect to y, such that

$$\begin{cases} \hat{P}_\varepsilon(x \to q) := \displaystyle\int_{\mathbb{R}^d} dy \exp\{iq\cdot y\}\,\bar{P}_\varepsilon(x \to y) \\ \hat{P}_0(x \to q) = n_0(x)\exp\{iq\cdot x\}\,. \end{cases} \tag{S.7}$$

When Eq.(S.6) is substituted into Eq.(S.6), we have

$$\frac{\partial}{\partial \varepsilon}\hat{P}_\varepsilon(x \to q) = -\int_{\mathbb{R}^d} dy \exp\{iq\cdot y\}\,\mathcal{H}'_{\varepsilon,y}\bar{P}_\varepsilon(x \to y)\,. \tag{S.8}$$

On the RHS, integration by parts is applied, such that

$$\begin{cases} -\displaystyle\int_{\mathbb{R}^d} dy \exp\{iq\cdot y\}\,\mathcal{H}'_{\varepsilon,y}\tilde{P}_\varepsilon(y) = -\int_{\mathbb{R}^d} dy \exp\{iq\cdot y\}\,\mathcal{H}'(\varepsilon,-iq,y)\,\tilde{P}_\varepsilon(x \to y) \\ \mathcal{H}'(\varepsilon,-iq,y) = -i\displaystyle\sum_{j=1}^{d} q^j\bar{g}_j(\varepsilon,y) + \frac{1}{2}\sum_{j,j'=1}^{d} q^j q^{j'}\bar{c}_{j,j'}(\varepsilon,y) + \bar{\mu}(\varepsilon,y)\,, \end{cases} \tag{S.9}$$

and expand $\bar{g}(\varepsilon,y)$, $\bar{c}_{j,j'}(\varepsilon,y)$, and $\bar{\mu}(\varepsilon,y)$ into a power series with respect to ε as follows:

$$\begin{cases} \bar{g}_j(\varepsilon,y) = \bar{g}_j(0,x) + \dfrac{\partial}{\partial \varepsilon}\bar{g}_j(\varepsilon,y)\Big|_{\varepsilon=0}\varepsilon + \nabla\bar{g}_j(\varepsilon,y)\cdot\dfrac{d}{d\varepsilon}y\Big|_{\varepsilon=0}\varepsilon + O(\varepsilon^2) \\ \bar{c}_{j,j'}(\varepsilon,y) = \bar{c}_{j,j'}(0,y) + \dfrac{\partial}{\partial \varepsilon}\bar{c}_{j,j'}(\varepsilon,y)\Big|_{\varepsilon=0}\varepsilon + \nabla\bar{c}_{j,j'}(\varepsilon,y)\cdot\dfrac{d}{d\varepsilon}y\Big|_{\varepsilon=0}\varepsilon + O(\varepsilon^2) \\ \bar{\mu}(\varepsilon,y) = \bar{\mu}(0,x) + \dfrac{\partial}{\partial \varepsilon}\bar{\mu}(\varepsilon,y)\Big|_{\varepsilon=0}\varepsilon + \nabla\bar{\mu}(\varepsilon,y)\cdot\dfrac{d}{d\varepsilon}y\Big|_{\varepsilon=0}\varepsilon + O(\varepsilon^2)\,. \end{cases} \tag{S.10}$$

Substituting (S.10) into (S.9), we obtain a transition rate for the sufficiently short time, ε, given by

$$\begin{aligned} \int_{\mathbb{R}^d} dy \exp\{iq\cdot y\}\,\mathcal{H}'_{\varepsilon,y}\bar{P}_\varepsilon(x \to y) &= \left(-\mathcal{H}'(0,-iq,y) + O(\varepsilon)\right)\hat{P}_\varepsilon(x \to q) \\ &\approx -\mathcal{H}'(0,-iq,y)\,\hat{P}_\varepsilon(x \to q)\,, \end{aligned} \tag{S.11}$$

where

$$\mathcal{H}'(0,-iq,y) = -i\sum_{j=1}^{d} q^j\bar{g}_j(0,x) + \frac{1}{2}\sum_{j,j'=1}^{d} q^j q^{j'}\bar{c}_{j,j'}(0,y) + \bar{\mu}(0,x)\,.$$

Substituting (S.11) into (S.8) and solving the ODE, we obtain the solution which is

$$\hat{P}_{\Delta\varepsilon}(x \to q) = n_0(x)\exp\{iq\cdot x - \mathcal{H}'(0,-iq,y)\,\Delta\varepsilon\}\,.$$

Using inverse transform of the above equation, $\bar{P}_{\Delta\varepsilon}(x\to y)$ becomes

$$\bar{P}_{\Delta\varepsilon}(x\to y)=\frac{n_0(x)}{(2\pi)^d}\int_{\mathbb{R}^d}dq\exp\{-iq\cdot(y-x)-\mathcal{H}'(0,-iq,x)\Delta\varepsilon\}. \tag{S.12}$$

Then, we set

$$\bar{K}'_{\Delta\varepsilon}(x\to y):=\frac{1}{(2\pi)^d}\int_{\mathbb{R}^d}dq\exp\{-iq\cdot(y-x)-\mathcal{H}'(0,-iq,x)\Delta\varepsilon\}, \tag{S.13}$$

and from being Markovian process in the dynamics at finite time, we show

$$\begin{aligned}\bar{K}_\varepsilon(x\to y)&=\int\cdots\int_A\prod_{\tau=1}^{T-1}dx_\tau\bar{K}'_{\Delta\varepsilon}(x_\tau\to x_{\tau+1})\\&=\frac{1}{(2\pi)^{d\times T}}\int\cdots\int_A\prod_{\tau=1}^{T-1}dx_\tau\int\cdots\int_{\mathbb{R}^d}\prod_{\tau=0}^{T-1}dq_\tau\\&\quad\times\exp\{-iq_\tau\cdot(x_{\tau+1}-x_\tau)-\mathcal{H}'(\varepsilon_\tau,-iq_\tau,x_\tau)\Delta\varepsilon\},\end{aligned} \tag{S.14}$$

where $\Delta\varepsilon=\varepsilon_{\tau+1}-\varepsilon_\tau\quad(\varepsilon_{\tau+1}>\varepsilon_\tau>0)$ and $\varepsilon_0=0$. It takes the limit of $\Delta\varepsilon$ to zero, then $T\Delta\varepsilon$ conserves a constant ε such that

$$\begin{aligned}&\lim_{\Delta\varepsilon\to0}\frac{1}{(2\pi)^{d\times T}}\int\cdots\int_A\prod_{\tau=1}^{T-1}dx_\tau\int\cdots\int_{\mathbb{R}^d}\prod_{\tau=0}^{T-1}dq_\tau\\&\quad\times\exp\{-iq_\tau\cdot(x_{\tau+1}-x_\tau)-\mathcal{H}'(\varepsilon_\tau,-iq_\tau,x_\tau)\Delta\varepsilon\}\Big|_{T\Delta\varepsilon=\varepsilon}.\end{aligned} \tag{S.15}$$

Accordingly, the limiting function expresses the summation over every projection function of stage transition which connect x with y at time ε, and that is the extended path-integral. We rewrite (S.15) as

$$\begin{aligned}\bar{K}_\varepsilon(x\to y)&=\int_{\tilde{X}_0=x}^{\tilde{X}_\varepsilon=y}\mathcal{D}(x)\int_{\mathbb{R}}\mathcal{D}(q)\exp\left\{\int_0^\varepsilon d\tau\left(-iq_\tau\cdot\dot{\tilde{X}}_\tau-\mathcal{H}'\left(\tau,-iq_\tau,\tilde{X}_\tau\right)\right)\right\}\\\mathcal{H}\left(\tau,-iq_\tau,\tilde{X}_\tau\right)&:=-\sum_{j=1}^d iq_{j,\tau}g_j\left(\tau,\tilde{X}_\tau\right)+\frac{1}{2}\sum_{j,j'=1}^d q_{j,\tau}q_{j',\tau}\bar{c}_{j,j'}\left(\tau,\tilde{X}_\tau\right)+\mu\left(\tau,\tilde{X}_\tau\right),\end{aligned} \tag{S.16}$$

where $\dot{\tilde{X}}_\tau$ represents the differential of $\tilde{X}_\tau$ with respect to τ and where

$$\int_{\tilde{X}_0=x}^{\tilde{X}_\varepsilon=y}\mathcal{D}(x)\int_{\mathbb{R}^d}\mathcal{D}(q):=\lim_{\Delta\varepsilon\to0}\frac{1}{(2\pi)^{d\times T}}\int\cdots\int_A\prod_{\tau=1}^{T-1}dx_\tau\int\cdots\int_{\mathbb{R}^d}\prod_{\tau=0}^{T-1}dq_\tau.$$

Taking $t>a$ into account and changing the original coordinate, (t,a), to the new coordinate, $(t-a,0)$ in Eq.(S.16), we obtain

$$P_{t-a}(\varepsilon,x\to y)=n_{t-a}(x)\tilde{K}_\varepsilon(x\to y).$$

Setting $\varepsilon=a$, we consequently have the path-integral expression of Eq.(S.4) as follows:

$$P_t(a,x\to y)=n_{t-a}(x)\tilde{K}_a(x\to y).$$

We then set

$$\lim_{a\downarrow0}\tilde{K}_a(x\to y):=\delta^d(x-y).$$

Substantially, let $\bar{\mathbf{C}}_\tau := \left(\bar{c}_{j,j'}\left(\tau, \tilde{X}_\tau\right)\right)$ be a $d \times d$ non-negative symmetric matrix and $\tilde{g}\left(a, \tilde{X}_a\right) := \left(\tilde{g}_1\left(a, X_a\right), \cdots, \tilde{g}_j\left(a, \tilde{X}_a\right), \cdots, \tilde{g}_d(a, X_a)\right)$ be a vector to simplify notations and to derive another expression of the projection function. The Hamiltonian, then, can be written as a quadratic form, such that

$$\mathcal{H}\left(\tau, -iq_\tau, \tilde{X}_\tau\right) = -iq_\tau \cdot \tilde{g}\left(\tau, \tilde{X}_\tau\right) + \frac{1}{2} q_\tau \cdot \bar{\mathbf{C}}_\tau q_\tau + \tilde{\mu}\left(\tau, \tilde{X}_\tau\right).$$

Note that terms of the RHS including q in Eq.(S.13) is identical to a characteristic function of multi-dimensional normal distribution, hence those terms represent the Fourier transform of the distribution. Consequently, another expression (the Lagrangian expression) is composed of the inverse transform of Eq.(S.13) and which is

$$\begin{aligned} \tilde{K}_a\left(x \to y\right) &= \int_{\tilde{X}_0 = x}^{\tilde{X}_a = y} \mathcal{D}\left(x\right) \frac{1}{Z'_\tau} \exp\left\{\int_0^a d\tau \mathcal{L}\left(\tau, \dot{\tilde{X}}_\tau, \tilde{X}_\tau\right)\right\} \\ \mathcal{L}\left(\tau, \dot{\tilde{X}}_\tau, \tilde{X}_\tau\right) &:= -\frac{1}{2}\left(\dot{\tilde{X}}_\tau - \tilde{g}\left(\tau, \tilde{X}_\tau\right)\right) \cdot \bar{\mathbf{C}}_\tau^{-1}\left(\dot{\tilde{X}}_\tau - \tilde{g}\left(\tau, \tilde{X}_\tau\right)\right) - \tilde{\mu}\left(\tau, \tilde{X}_\tau\right), \end{aligned} \tag{S.17}$$

where Z'_τ denotes normalization constant. This expression is used in the analysis of classical state transition curve (no stochasticity) [3].

Correspondence of path-integral formulation to a TMM

We show a TMM corresponding to Eq.(22) at $d = 1$ and $N = 1$. Let

$$\begin{aligned} \mathbf{a} &:= (0, 1, 2, \cdots, a, \cdots, \alpha) \\ \mathbf{y} &:= (y_0, y_1, y_2, \cdots, y_m, \cdots, y_M) \end{aligned}$$

and ω be the age vector, the size-vector, and $\omega = \alpha(M+1)+1$, respectively. Setting a compact set $\hat{A} \in \mathbb{R}_+$ and size interval h dividing $\hat{A}$ into M categories, y_m represents m-th size category. We can show the following $\omega \times \omega$ TMM:

$$\mathbf{P}_{t+1}\left(\mathbf{a}, \mathbf{y}\right) = \mathbf{L}\mathbf{P}_t\left(\mathbf{a}, \mathbf{y}\right) \tag{S.18}$$

where

$$\mathbf{L} = \begin{bmatrix} f_0 & \mathbf{F} & \cdots & \mathbf{F} & \cdots & \mathbf{F} \\ \mathbf{K}_0 & O & \cdots & O & \cdots & O \\ 0 & \mathbf{K}_1 & \cdots & O & \cdots & O \\ \vdots & O & \ddots & O & \cdots & O \\ 0 & \cdots & O & \cdots & \mathbf{K}_{\alpha-1} & O \end{bmatrix}, \tag{S.19}$$

in $d = 1$ and $N = 1$. This TMM is common with Leslie matrix on the mathematical form, however, each vital rate is composed of the following matrices: $\mathbf{F}$, O, and $\mathbf{K}_a$ are $\mathbf{F}_a = \begin{pmatrix} f_0 & \cdots & f_m & \cdots & f_M \end{pmatrix}$, $(M+1) \times (M+1)$ zero matrix, and $(M+1) \times (M+1)$ state transition matrix if and only if $\mathbf{K}_0 := \left(k_{0,0}, \cdots, k_{0,m}, \cdots, k_{0,M}\right)^{\mathrm{T}}$, respectively. Focusing on two elements of the population vector,

$$P_{t+1}\left(a+1, y_m\right) = \sum_{m'=0}^{M} k_{a,m'm} P_t\left(a, y_{m'}\right) \tag{S.20a}$$

$$P_{t+1}\left(0, y_0\right) = \sum_{a=0}^{\alpha} \sum_{m=0}^{M} f_m P_t\left(a, y_m\right) \tag{S.20b}$$

The first recursion, Eq.(S.20a), is formally solvable and the cohort can be expressed by

$$P_t(a, y_m) = \sum_{m'=0}^{M} \sum_{m''=0}^{M} \cdots \sum_{m^{(a)}=0}^{M} \overbrace{k_{a-1,m'm} k_{a-2,m''m'} \cdots k_{1,m^{(a)}m^{(a-1)}} k_{0,0m^{(a)}}}^{a \text{ times}} P_{t-a}(0, y_0). \tag{S.21}$$

The RHS of Eq.(S.21) describes the sum over all possible transitions of life history starting from initial state $P_{t-a}(0, y_0)$ to $P_t(a, y_m)$. In other word, one expects similar relationship between Eq.(19) and Eq.(S.21) in the concept of path-integral as well. Since each vital rate of $\mathbf{K}$ generally includes survival rate $(p_{a,m})$, we decompose the vital rate $k_{a,m'm}$ to a pure transition probability $k'_{a,m'm}$ and the survival rate as follows:

$$k_{a,m'm} = k'_{a,m'm} \times p_{a,m'} \tag{S.22}$$

Substituting this into Eq.(S.20b) and assuming Y_a to be a stochastic process of size growth generated by the following parameterized collection of random variables Eq.(27),

$$\{Y_a\}_{a\in[0,\alpha]} := \{y_{a,m(a)}\}_{a\in[0,\alpha]}, \qquad (m(0) = 0) \tag{S.23}$$

we can rewrite the equation as follows:

$$\begin{aligned} P_{t+1}(0, y_0) &= \sum_{a=0}^{\alpha} \sum_{m\in\hat{A}} f_m P_t(a, y_m) \\ &= \sum_{a=0}^{\alpha} \sum_{m(a)\in\hat{A}} \cdots \sum_{m(0)\in\hat{A}} f_{m(a)} \times \prod_{\tau=0}^{a-1} p_{\tau,m(\tau)} k'_{\tau,m(\tau)m(\tau+1)} P_{t-a}(0, y_0) \\ &= \sum_{a=0}^{\alpha} \mathbb{E}_{y_0}[f(Y_a) S(a)] P_{t-a}(0, y_0), \end{aligned} \tag{S.24}$$

where components are

$$f(Y_a) = f_{m(a)}, \qquad S(a) = \prod_{\tau=0}^{a-1} p(\tau, Y_\tau) = \prod_{\tau=0}^{a-1} p_{\tau,m(\tau)}.$$

When $P^*(0, y_0)$ is the first state in eigenvector of $\mathbf{L}$, the above equation is rewritten by using the eigenvalue, λ_0, as follows:

$$P^*(0, y_0) = \sum_{a=0}^{\alpha} \mathbb{E}_{y_0}[f(Y_a) S(a)] \lambda_0^{-a-1} P^*(0, y_0). \tag{S.25}$$

Since $P^*(0, y_0)$ of both sides can be canceled, we have the characteristic equation of $\mathbf{L}$

$$1 = \sum_{a=0}^{\alpha} \mathbb{E}_{y_0}[f(Y_a) S(a)] \lambda_0^{-a-1}. \tag{S.26}$$

This characteristic equation obviously parallels Eq.(9), and is nothing but the generalized Euler–Lotka equation in TMM to age-size model. Furthermore, $k_{a,m',m}$ is assumed to follow Gaussian distribution

associated with mortality at an age interval Δa, such that

$$\begin{aligned} k_{a,m',m} &\equiv h\bar{K}_{\Delta a}\left(y_{m'}\to y_m\right) \\ &:= \frac{h}{\left(2\pi\sigma_{1,1}\left(y_{m'}\right)^2\Delta a\right)^{\frac{1}{2}}}\times \\ &\exp\left\{\left[-\frac{1}{2}\frac{\left(\frac{y_m-y_{m'}}{\Delta a}-g_1\left(y_{m'},v\right)\right)^2}{\sigma_{1,1}\left(y_{m'}\right)^2}-\mu\left(y_{m'},v\right)\right]\Delta a\right\}. \end{aligned} \tag{S.27}$$

Rewriting Eq.(S.27) for the characteristic function form, we have

$$\begin{aligned} \bar{K}_{\Delta a}\left(y_{m'}\to y_m\right) &= \frac{1}{2\pi}\int_{-\infty}^{\infty}dq^1\exp\left\{-iq^1\left(\frac{y_m-y_{m'}}{\Delta a}\right)\Delta a-\mathcal{H}'\left(-iq^1,y_{m'}\right)\Delta a\right\} \\ \mathcal{H}'\left(-iq^1,y_{m'}\right) &:= -iq^1 g_1\left(y_{m'},v\right)+\frac{1}{2}\left(q^1\right)^2\sigma_{1,1}\left(y_{m'},v\right)+\mu\left(y_{m'},v\right). \end{aligned} \tag{S.28}$$

We can adopt those transition rates to the projection function as follows:

$$n_{t-a}\left(x\right)K_a\left(x\to y\right)=\lim_{h,\Delta a\downarrow 0}\sum_{m(a)\in A}\cdots\sum_{m(0)\in A}\prod_{\tau=0}^{a-1}k_{\tau,m(\tau)m(\tau+1)}P_{t-a}\left(0,y_0\right), \tag{S.29}$$

where variables are assumed by $y_{m(a)}=y$ and $y_0=x$. On the other hand, setting f_m be $f_m\equiv f_m\left(\Delta a\right)=\Delta aF\left(y_m\right)$, Eqs.(22) and (S.24) have the following relationship,

$$\int_0^{\alpha}da\ n_{t-a}\left(x\right)u_a\left(x\right)=\lim_{h,\Delta a\downarrow 0}\sum_{a=0}^{\alpha}\mathbb{E}_{y_0}\left[f\left(Y_a\right)S\left(a\right)\right]P_{t-a}\left(0,y_0\right). \tag{S.30}$$

Our model is on the hypothesis that difference of vital rates yield to the Gaussian distribution at infinitesimal short age interval Δa. Consequently, path-integral model is an expression of LDMs in the sense that one of expression has correspondence with those of the others if the temporal development of life histories can be approximated by local Gaussian process (Ito process), such as Eq.(1).

S2

Let $\tilde{v}$ be

$$\tilde{v}\in\mathcal{V},\quad \text{such that}\quad \lambda^{*,\tilde{v}}=\sup_v\lambda^{*,v},$$

and $\psi_\lambda^v\left(x\right)$ is given by Eq.(9). Define $\tilde{\lambda}$ by

$$\tilde{\lambda}:=\lambda^{*,\tilde{v}}.$$

Then, we have

$$\psi_{\tilde{\lambda}}^v\left(x\right)\leq\psi_{\tilde{\lambda}}^{\tilde{v}}\left(x\right)\Leftrightarrow\lambda^{*,v}\leq\lambda^{*,\tilde{v}}.$$

Proof. The key point is that $\psi_\lambda^v\left(x\right)$ is monotonically decreasing in λ with

$$\psi_{\lambda^{*,v}}\left(x\right)=1.$$

Therefore,

$$\psi_{\tilde{\lambda}}^v\left(x\right)\leq\psi_{\lambda^{*,v}}^v\left(x\right)=1=\psi_{\tilde{\lambda}}^{\tilde{v}}\left(x\right),$$

and the result follows trivially. □

S3

Viscosity solution is an important idea to consider Hamiltonian systems and is introduced in 80's [4]. The idea unifies Hamiltonian in population vector and in control theory.

Definition of viscosity solutions

We set the function

$$H : [0, \alpha) \times A \times \mathbb{R}^d \times \mathbb{S}^d \times \mathbb{R}_+ \mapsto \mathbb{R},$$

where $\mathbb{S}^d$ denotes a set of all real symetric matrices. When H is degenerate elliptic in E, it satisfies

$$Q_1 \leq Q_2 \rightarrow H(a, x, p, Q_1, w) \geq H(a, x, p, Q_2, w),$$

where $a \in [0, \alpha)$, $x \in A$, $p \in \mathbb{R}^d$, $Q \in \mathbb{S}^d$, and $w \in \mathbb{R}_+$. If the function is a monotonic function in w, it satisfies

$$w_1 \leq w_2 \rightarrow H(a, x, p, Q, w_1) \leq H(a, x, p, Q, w_2).$$

For example, Hamiltonian

$$H_0(x, p, Q, w) = \inf_v \left\{ -\sum_j^d g_j(x, v) p_j - \frac{1}{2} \sum_{j,j'=1}^d c_{j,j'}(x, v) Q_{j,j'} + [\mu(y, v) + \lambda] w \right\} - F(x), \tag{S3.1}$$

is degenerate elliptic, monotonically increasing function. This function is the same Hamiltonian used in the stochastic maximum principle [5]. Then, p, Q, and w represent co-state variables in the principle. Let H be degenerate elliptic and monotonic function. A sub-solution in the viscosity sense, $\psi \in C(A)$, of

$$H\left(x, D\psi, D^2\psi, \psi\right) = 0, \tag{S3.2}$$

is defined by $\psi - \hat{\psi}$ ($\hat{\psi} \in C^2(A)$) having a maximum value of zero at $\bar{x}$ and $\hat{\psi}$ satisfying

$$H\left(x, D\hat{\psi}, D^2\hat{\psi}, \hat{\psi}\right)\Big|_{x=\bar{x}} \leq 0. \tag{S3.3}$$

We then use abbreviation of derivatives:

$$D\psi := \left(\frac{\partial}{\partial x^1}\psi, \cdots, \frac{\partial}{\partial x^j}\psi, \cdots, \frac{\partial}{\partial x^d}\psi\right); D^2\psi := \left(\frac{\partial^2}{\partial x^j \partial x^{j'}}\psi\right).$$

A super-solution of Eq.(S3.2) in the viscosity sense, $\psi \in C(A)$, is defined by $\psi - \hat{\psi}$ ($\hat{\psi} \in C^2(A)$). It has a minimal value of zero at $\bar{x}$ and $\hat{\psi}$ satisfying

$$H\left(x, D\hat{\psi}, D^2\hat{\psi}, \hat{\psi}\right)\Big|_{x=\bar{x}} \geq 0. \tag{S3.4}$$

When ψ satisfies the sub- and super-solution in the viscosity sense, it is referred to as a "viscosity solution" [6]. Additionally, the viscosity solution of the nonlinear evolutional PDE can be defined as the sub-solution of

$$-\frac{\partial}{\partial a}w + H\left(a, x, Dw, D^2w, w\right) = 0 \tag{S3.5}$$

in the viscosity sense. $w \in C([0, \alpha) \times A)$ is defined by $w - \hat{w}$ ($\hat{w} \in C^{1,2}([0, \alpha) \times A)$), having a maximum value of zero at $(\bar{a}, \bar{x})$ and $\hat{w}$ satisfying

$$-\frac{\partial}{\partial a}\hat{w}\Big|_{a=\bar{a},x=\bar{x}} + H\left(a, x, D\hat{w}, D^2\hat{w}, \hat{w}\right)\Big|_{a=\bar{a},x=\bar{x}} \leq 0. \tag{S3.6}$$

A super-solution of Eq.(S3.5) in the viscosity sense, $w \in C([0,\alpha)\times A)$, is defined by $w-\hat{w}$ ($\hat{w} \in C^{1,2}([0,\alpha)\times A)$) having a minimum value of zero at $(\bar{a},\bar{x})$ and $\hat{w}$, satisfying

$$-\frac{\partial}{\partial a}\hat{w}\Big|_{a=\bar{a},x=\bar{x}} + H\left(a,x,D\hat{w},D^2\hat{w},\hat{w}\right)\Big|_{a=\bar{a},x=\bar{x}} \geq 0. \tag{S3.7}$$

When w satisfies the sub-solution and the super-solution of the above PDE in the viscosity sense, it is called a "viscosity solution" of the PDE.

Extension of the solutions by viscosity solutions unifies two approaches of analysis involved in the HJB equation and the maximum principle in control theory [7]. One can then find the correspondence of (p,Q,w) to $\left(D\hat{\psi},D^2\hat{\psi},\hat{\psi}\right)$ in Eq.(S3.1). The existence of co-state variables and the value function are important for optimal control over both the maximum principle and HJB equations approach. Focusing on the first, the second, and the third terms of the Hamiltonians in Eqs.(S3.1), Eq.(19), (37), and (46), they all have a common functional form: $H(x,p,Q,w)$, $H\left(x,iq_\tau,(iq_\tau)^2,(iq_\tau)^0\right)$, $H\left(x,D\tilde{\psi},D^2\tilde{\psi},\tilde{\psi}\right)$ and $H\left(x,D\tilde{w},D^2\tilde{w},\tilde{w}\right)$ with respect to each costate variable, respectively. $H\left(x,iq_\tau,(iq_\tau)^2,(iq_\tau)^0\right)$ shows that this Hamiltonian is the conjugate of the Hamiltonian in the path integral, Eq.(19), with respect to $-iq_\tau$. Consequently, the fittest has a minimum Hamiltonian in its habitat, and the Hamiltonian naturally appears in our formulation.

S4

Derivation of a general $\psi^v_\lambda(x)$ in semelparous species

Since X_a has strong Markov property that is a property of Ito's SDE [1], we can show the following relationship:

$$\begin{aligned}
\psi_{\mathcal{S}_{\lambda,x^*}}(x) &= \int_0^\infty da\exp\{-\lambda a\}\mathbb{E}_x\left[F_{\mathrm{S}}(X_a)S_{\mathrm{S}}(a)\right]\\
&= \mathbb{E}_x\left[\int_0^\infty da\exp\{-\lambda a\}F_{\mathrm{S}}(X_a)S_{\mathrm{S}}(a)\right]\\
&= \mathbb{E}_x\left[\int_{a^*}^\infty da\exp\{-\lambda a\}F_{\mathrm{S}}(X_a)S_{\mathrm{S}}(a)\right] \leftarrow \text{because of Eq.(4)}\\
&= \mathbb{E}_x\left[\exp\{-\lambda a^*\}\int_0^\infty d\tau\exp\{-\lambda\tau\}F_{\mathrm{S}}(X_{\tau+a^*})S_{\mathrm{S}}(\tau+a^*)\right]\\
&= \mathbb{E}_x\left[\exp\{-\lambda a^*\}S_{\mathrm{S}}(a^*)\int_0^\infty d\tau\exp\{-\lambda\tau\}F_{\mathrm{S}}(X_\tau)S_{\mathrm{S}}(\tau)\right]\\
&= \mathbb{E}_x\left[\exp\{-\lambda a^*\}S_{\mathrm{S}}(a^*)\right]\mathbb{E}^v_{x^*}\left[\int_0^\infty d\tau\exp\{-\lambda\tau\}F_{\mathrm{S}}(X_\tau)S_{\mathrm{S}}(\tau)\right]\\
&= \mathbb{E}_x\left[\exp\{-\lambda a^*\}S_{\mathrm{S}}(a^*)\right]\phi(x^*). \quad \uparrow \text{using strong Markov property}
\end{aligned}$$

S5

Mature age density of semelparous species

Since the optimal utilization is constant, we can use an adjoint Hamiltonian generated by geometric Brownian motion as follows:

$$\bar{\mathcal{H}}_x = -\gamma_0 x\frac{\partial}{\partial x} - \frac{\sigma_0^2}{2}x^2\frac{\partial^2}{\partial x^2} + \mu_0, \tag{S5.1}$$

where $\gamma_0 = \gamma_1 (1-v) + \gamma_2 v$ and $\sigma_0^2 = (\sigma_1 (1-v))^2 + (\sigma_2 v)^2$. From the expression of mature age density in Eq.(51), $u_a(x)$ and $\psi_0(x)$ satisfies

$$\begin{cases} \frac{\partial}{\partial a} u_a(x) = -\bar{\mathcal{H}}_x u_a(x) \\ u_0(x) = F_{\mathrm{S}}(x) \\ -\bar{\mathcal{H}}_x \psi_0(x) + F_{\mathrm{S}}(x) = 0. \end{cases} \tag{S5.2}$$

Setting a new variable $z = \log x$ and $u_a(x)$, such that

$$u_a(x) = \exp\left\{-\left(\gamma_0 - \frac{\sigma_0^2}{2}\right)\left[z + \left(\gamma_0 - \frac{\sigma_0^2}{2}\right)a/2\right]/(\sigma_0^2) - \mu_0 a\right\} w_a(z), \tag{S5.3}$$

the equation of $u_a(x)$ in Eq.(S5.1) becomes

$$\begin{cases} \frac{\partial}{\partial a} w_a(z) = \frac{\sigma_0^2}{2}\frac{\partial^2}{\partial z^2} w_a(z) \\ \quad w_0(z) = \exp\left\{\left(\gamma_0 - \frac{\sigma_0^2}{2}\right) z/(\sigma_0^2)\right\} F_{\mathrm{S}}(\exp\{z\}) \end{cases} \tag{S5.4}$$

This means, therefore, that statistics of Eq.(59) come down to those of Brownian motion via the above heat equation. The mature age density then is converted to the first passage time problem of the Brownian motion. When converted mature size is $z^* := \log x^*$, the probabilty of $B_a > z^*$, $\mathbb{P}_z(B_a > z^*)$, satisfies the following equation by using the fundamental solution in Eq.(S5.4)

$$\begin{aligned} 2\mathbb{P}_z(B_a > z^*) &= \int_{z^*}^{\infty} d\zeta \frac{2}{\sqrt{2\pi\sigma_0^2 a}} \exp\left\{-\frac{(\zeta - z)^2}{2\sigma_0^2 a}\right\} \\ &= \sqrt{\frac{2}{\pi}} \int_{\frac{z^*-z}{\sqrt{\sigma_0^2 a}}}^{\infty} d\zeta' \exp\left\{-\frac{\zeta'^2}{2}\right\}. \end{aligned} \tag{S5.5}$$

If $a^*_{z^*}$ denotes the first passage time of z^*, it is known as "reflection principle" [2] that

$$\mathbb{P}_z(a^*_{z^*} < a) = 2\mathbb{P}_z(B_a > z^*)$$

We, then, obtain $\mathbb{P}_z(a^*_{z^*} \in da)$ by differentiating both sides with respect to a as follows:

$$\mathbb{P}_z(a^*_{z^*} \in da) = \frac{z^* - z}{\sqrt{2\pi\sigma_0^2 a^3}} \exp\left\{-\frac{(z^* - z)^2}{2\sigma_0^2 a}\right\} da \tag{S5.6}$$

This fuction called inverse Gaussian distribution [8]. From Eqs.(44), (51), $z^* := \log x^*$, $z^* := \log x^*$, and Eq.(S5.3), the mature age density in original parameters becomes

$$\mathbb{A}(a) = \frac{1}{\mathbb{E}_x[S(a^*)]} \frac{\log\frac{x^*}{x}}{\sqrt{2\pi\sigma_0{}^2 a^3}} \exp\left\{-\frac{\left[\log\frac{x^*}{x} - \left(\gamma_0 - \frac{\sigma_0{}^2}{2}\right)a\right]^2}{2\sigma_0{}^2 a} - \mu_0 a\right\}, \tag{S5.7}$$

where $\mathbb{E}_x[S(a^*)] = \psi_0(x)/\phi(x^*)$. In other words, the expectation is the solution of

$$-\bar{\mathcal{H}}_x \mathbb{E}_x[S(a^*)] + \mathbf{1}_{\{x=x^*\}} = 0. \tag{S5.8}$$

Therefore, that is

$$\begin{aligned}\mathbb{E}_x\left[S\left(a^*\right)\right] &= \left(\frac{x}{x^*}\right)^{\rho_0} \\ \rho_0 &= \frac{1}{2}\left(1-\frac{2\gamma}{{\sigma_0}^2}\right)+\frac{1}{2}\sqrt{\left(1-\frac{2\gamma}{{\sigma_0}^2}\right)^2+\frac{8\mu_0}{{\sigma_0}^2}}\end{aligned} \tag{S5.9}$$

Consequently, the semelparous mature age density that all parameters appear follows

$$\begin{aligned}&\mathbb{A}_{\mathrm{S}}\left(a\right) = \\ &= \frac{\log\frac{x^*}{x}}{\sqrt{2\pi\left[\sigma_1^2v^2+\sigma_2^2\left(1-v\right)^2\right]a^3}} \\ &\quad\times\exp\left\{-\frac{\left[\log\frac{x^*}{x}-\left(\left[\gamma_1\left(1-v\right)+\gamma_2v\right]-\frac{1}{2}\left[\sigma_1^2v^2+\sigma_2^2\left(1-v\right)^2\right]\right)a\right]^2}{2\left[\sigma_1^2v^2+\sigma_2^2\left(1-v\right)^2\right]a}+\rho_v\log\frac{x^*}{x}-\mu_0a\right\} \\ &\rho_v := \frac{1}{2}\left(1-\frac{2\left[\gamma_1\left(1-v\right)+\gamma_2v\right]}{\sigma_1^2v^2+\sigma_2^2\left(1-v\right)^2}\right) \\ &\quad+\frac{1}{2}\sqrt{\left(1-\frac{2\left[\gamma_1\left(1-v\right)+\gamma_2v\right]}{\sigma_1^2v^2+\sigma_2^2\left(1-v\right)^2}\right)^2+\frac{8\mu_0}{\sigma_1^2v^2+\sigma_2^2\left(1-v\right)^2}}.\end{aligned} \tag{S5.10}$$

References

[1] Øksendal B (2003) Stochastic differential equations: an introduction with applications. Springer Verlag.

[2] Karatzas I, Shreve S (1991) Brownian motion and stochastic calculus, volume 113. Springer Verlag.

[3] Oizumi R, Takada T (2013) Optimal life schedule with stochastic growth in age-size structured models: theory and an application. Journal of Theoretical Biology 323: 76–89.

[4] Crandall MG, Lions PL (1983) Viscosity solutions of hamilton-jacobi equations. Trans Amer Math Soc 277: 1–42.

[5] Yong J, Zhou X (1999) Stochastic controls: Hamiltonian systems and HJB equations, volume 43. Springer Verlag.

[6] Crandall MG, Ishii H, Lions PL (1992) User ' s guide to viscosity solutions of second order partial differential equations. Bull Amer Math Soc 27: 1–67.

[7] Peng S (1992) A generalized dynamic programming principle and hamilton-jacobi-bellman equation. Stochastics: An International Journal of Probability and Stochastic Processes 38: 119–134.

[8] Letac G, Seshadri V (1983) A characterization of the generalized inverse gaussian distribution by continued fractions. Probability Theory and Related Fields 62: 485–489.
